# Supplementary material for: Effect of Multi-Nutrient Milk Fortification on Preterm Neonate Outcomes: A Network Meta-Analysis
Source: Nutrients. 2025 May 12;17(10):1651. doi: 10.3390/nu17101651 (PMC12113778; doi:10.3390/nu17101651)
Supplement: Supplementary file 1 [file nutrients-17-01651-s001.zip › nutrients-3552039-supplementary.pdf]

Table of Contents

Supplementary Materials 2
1.0 Methods 2
1.1 Search strategy 2
1.2 Description of changes from protocol 5
1.3 Priors 5
1.4 Reproducibility 5
2.0 Results 12
2.1 References to trials included in the systematic review 12
2.2 Study characteristics 16
2.3 Risk of bias assessment 22
2.4 League tables 25

Supplementary Materials

S1.0 Methods

S1.1 Search strategy

MEDLINE Search Strategy:

Ovid MEDLINE(R) All
1. exp Infant, Premature/
2. exp Infant, Low Birth Weight/
3. ((prematur* or pre matur* or preterm* or pre term*) adj2 (infant* or newborn? or baby or babies or neonat* or neo nat* or child*)).tw.
4. (premie? or preemie?).tw.
5. (low adj2 (birthweight? or birth weight?)).tw.
6. (small adj2 gestation* age).tw.
7. or/1-6
8. Milk, Human/
9. Infant Formula/
10. (milk adj2 (breast* or human* or mother* or maternal* or woman* or women* or express* or artificial*)).tw.
11. breastmilk*.tw.
12. formula.tw.
13. or/8-12
14. Food, Fortified/
15. ((breast* or human* or mother* or maternal* or woman* or women* or express*) adj2 (fortif* or supplement*)).tw.
16. ((bovine* or cow* or cattle* or animal*) adj2 (fortif* or supplement*)).tw.
17. prolacta.tw.
18. h2mf.tw.
19. Milk Banks/
20. (donor? or donat* or bank*).tw.
21. or/14-20
22. 7 and 13 and 21

S1.1 Other Search Strategies:

	**CINAHL**
1	(MH "Infant, Premature")
2	(MH "Infant, Low Birth Weight+")
3	(prematur* or pre matur* or preterm* or pre term*) N2 (infant* or newborn# or baby or babies or neonat* or neo nat* or child*)
4	premie# or preemie#
5	low N2 (birthweight# or birth weight#)
6	small N2 (gestation* age)
7	S1 OR S2 OR S3 OR S4 OR S5 OR S6
8	(MH "Milk, Human")
9	(MH "Infant Feeding+")
10	(MH "Infant Formula")

	**Embase**
1	'prematurity'/exp
2	'low birth weight'/exp
3	(prematur* OR 'pre matur*' OR preterm* OR 'pre term*') NEAR/2 (infant* OR newborn* OR baby OR babies OR neonat* OR 'neo nat*' OR child*)
4	premie* OR preemie*
5	low NEAR/2 (birthweight* OR 'birth weight*')
6	small NEAR/2 'gestation* age'
7	#1 OR #2 OR #3 OR #4 OR #5 OR #6
8	'breast milk'/exp
9	'infant feeding'/exp
10	'artificial milk'/exp

	**Web of Science - NOT UPDATED IN 2023**
	no thesaurus in WoS
	no thesaurus in WoS
1	TS=((prematur* OR "pre matur*" OR preterm* OR "pre term*") NEAR/2 (infant* OR newborn* OR baby OR babies OR neonat* OR "neo nat*" OR child*))
2	TS=(premie* OR preemie*)
3	TS=(low NEAR/2 (birthweight* OR "birth weight*"))
4	TS=(small NEAR/2 "gestation* age")
5	#4 OR #3 OR #2 OR #1
	no thesaurus in WoS
	no thesaurus in WoS
	no thesaurus in WoS

	**CENTRAL**
1	MeSH descriptor: [Infant, Premature] explode all trees
2	MeSH descriptor: [Infant, Low Birth Weight] explode all trees
3	(prematur* or pre matur* or preterm* or pre term*) near/2 (infant* or newborn* or baby or babies or neonat* or neo nat* or child*)
4	premie* or preemie*
5	low near/2 (birthweight* or birth weight*)
6	small near/2 (gestation* age)
7	#1 or #2 or #3 or #4 or #5 or #6
8	MeSH descriptor: [Milk, Human] explode all trees
	-
9	MeSH descriptor: [Infant Formula] explode all trees

11	milk N2 (breast* or human* or mother* or maternal* or woman* or women* or express* or artificial*)	11	milk NEAR/2 (breast* OR human* OR mother* OR maternal* OR woman* OR women* OR express* OR artificial*)	6	TS=(milk NEAR/2 (breast* OR human* OR mother* OR maternal* OR woman* OR women* OR express* OR artificial*))	10	milk near/2 (breast* or human* or mother* or maternal* or woman* or women* or express* or artificial*)
12	breastmilk*	12	breastmilk*	7	TS=(breastmilk*)	11	breastmilk*
13	formula	13	formula	8	TS=(formula)	12	formula
14	S8 OR S9 OR S10 OR S11 OR S12 OR S13	14	#8 OR #9 OR #10 OR #11 OR #12 OR #13	9	#8 OR #7 OR #6	13	#8 or #9 or #10 or #11 or #12
15	(MH "Food, Fortified")	15	'fortified food'/exp		*no thesaurus in WoS*	14	MeSH descriptor: [Food, Fortified] explode all trees
16	(breast* or human* or mother* or maternal* or woman* or women* or express*) N2 (fortif* or supplement*)	16	(breast* OR human* OR mother* OR maternal* OR woman* OR women* OR express*) NEAR/2 (fortif* OR supplement*)	10	TS=((breast* OR human* OR mother* OR maternal* OR woman* OR women* OR express*) NEAR/2 (fortif* OR supplement*))	15	(breast* or human* or mother* or maternal* or woman* or women* or express*) near/2 (fortif* or supplement*)
17	(bovine* or cow* or cattle* or animal*) N2 (fortif* or supplement*)	17	(bovine* OR cow* OR cattle* OR animal*) NEAR/2 (fortif* OR supplement*)	11	TS=((bovine* OR cow* OR cattle* OR animal*) NEAR/2 (fortif* OR supplement*))	16	(bovine* or cow* or cattle* or animal*) near/2 (fortif* or supplement*)
18	prolacta	18	prolacta	12	TS=(prolacta)	17	prolacta
19	h2mf	19	h2mf	13	TS=(h2mf)	18	h2mf
20	(MH "Milk Banks")	20	'milk bank'/exp		*no thesaurus in WoS*	19	MeSH descriptor: [Milk Banks] explode all trees
21	(MH "Donor Milk")		-		*no thesaurus in WoS*		-
22	donor# or donat* or bank*	21	donor* OR donat* OR bank*	14	TS=(donor* OR donat* OR bank*)	20	donor* or donat* or bank*
23	S15 OR S16 OR S17 OR S18 OR S19 OR S20 OR S21 OR S22	22	#15 OR #16 OR #17 OR #18 OR #19 OR #20 OR #21	15	#14 OR #13 OR #12 OR #11 OR #10	21	#14 or #15 or #16 or #17 or #18 or #19 or #20
24	S7 AND S14 AND S23	23	#7 AND #14 AND #22	16	#15 AND #9 AND #5	22	#7 and #13 and #21

S1.2 Description of changes from the protocol

There were changes made to the reported outcomes due to project scope. The excluded outcomes were intracranial hemorrhage, length of stay in ICU, all neurodevelopment and neurosensory impairment measures except Bayley II MDI, all breastfeeding outcomes, and other neonatal illnesses considered to be clinically meaningful (e.g., diarrhea, lower respiratory tract disease). In addition, it was not possible to perform sub-group analysis due to the small sample size. Furthermore, no meta-regression analysis was conducted due to insufficient data. Lastly, inconsistency was not assessed due to the limited interactions, and therefore greater emphasis was placed on the qualitative assessment of intransitivity.

S1.3. Priors

Parameter	Binary outcomes	Continuous outcomes
Trial baseline fixed effect	Normal(0,10)	Normal(0, 100)
Basic parameters	Normal(Class mean, Class sd)	
Class mean	Normal(0, 1)	Normal(0, 10)
Class sd	Half-Normal(0,1) bound at 0	Half-Normal(0, 0.25)
Between trial sd	Uniform(0,2)	Uniform(0,2)

Normal distributions specified as mean and standard deviation. Note that JAGS models are specified in terms of mean and precision.

S1.4 Reproducibility

```
## ─ Session info ───────────────────────────────────────────
##  setting  value
##  version  R version 4.3.2 Patched (2024-01-03 r85769)
```

```
##  os       macOS Sonoma 14.2
##  system   aarch64, darwin20
##  ui       RStudio
##  language (EN)
##  collate  en_US.UTF-8
##  ctype    en_US.UTF-8
##  tz       America/Halifax
##  date     2024-01-18
##  rstudio  2023.12.0+369 Ocean Storm (desktop)
##  pandoc   3.1.1 @ /Applications/RStudio.app/Contents/Resources/app/quarto/bin/tools/ (via rmarkdown)
##
## ─ Packages ───────────────────────────────────────────────────
##  ! package           * version    date (UTC) lib source
##    abind               1.4-5      2016-07-21 [1] CRAN (R 4.3.0)
##    askpass             1.2.0      2023-09-03 [1] CRAN (R 4.3.0)
##    assertr           * 3.0.1      2023-11-23 [1] CRAN (R 4.3.1)
##    assertthat          0.2.1      2019-03-21 [1] CRAN (R 4.3.0)
##    aws.s3              0.3.21     2020-04-07 [1] CRAN (R 4.3.0)
##    aws.signature       0.6.0      2020-06-01 [1] CRAN (R 4.3.0)
##    backports           1.4.1      2021-12-13 [1] CRAN (R 4.3.0)
##    base64enc           0.1-3      2015-07-28 [1] CRAN (R 4.3.0)
##    boot                1.3-28.1   2022-11-22 [1] CRAN (R 4.3.2)
##    brio                1.1.4      2023-12-10 [1] CRAN (R 4.3.1)
##    broom             * 1.0.5      2023-06-09 [1] CRAN (R 4.3.0)
##    broom.helpers       1.14.0     2023-08-07 [1] CRAN (R 4.3.0)
##    cachem              1.0.8      2023-05-01 [1] CRAN (R 4.3.0)
##    cellranger          1.1.0      2016-07-27 [1] CRAN (R 4.3.0)
##    checkmate           2.3.1      2023-12-04 [1] CRAN (R 4.3.1)
##    cli                 3.6.2      2023-12-11 [1] CRAN (R 4.3.1)
##    coda                0.19-4     2020-09-30 [1] CRAN (R 4.3.0)
##    codetools           0.2-19     2023-02-01 [1] CRAN (R 4.3.2)
##    colorspace          2.1-0      2023-01-23 [1] CRAN (R 4.3.0)
##    CompQuadForm        1.4.3      2017-04-12 [1] CRAN (R 4.3.0)
##    crayon              1.5.2      2022-09-29 [1] CRAN (R 4.3.0)
##    crul                1.4.0      2023-05-17 [1] CRAN (R 4.3.0)
##    curl                5.2.0      2023-12-08 [1] CRAN (R 4.3.1)
```

```
##    data.table          1.14.10    2023-12-08 [1] CRAN (R 4.3.1)
##    data.validator    * 0.2.1      2023-12-11 [1] CRAN (R 4.3.1)
##    desc                1.4.3      2023-12-10 [1] CRAN (R 4.3.1)
##    devtools            2.4.5      2022-10-11 [1] CRAN (R 4.3.0)
##    DiagrammeR        * 1.0.10     2023-05-18 [1] CRAN (R 4.3.0)
##    diffobj             0.3.5      2021-10-05 [1] CRAN (R 4.3.0)
##    digest              0.6.33     2023-07-07 [1] CRAN (R 4.3.0)
##    dplyr             * 1.1.4      2023-11-17 [1] CRAN (R 4.3.1)
##    ellipsis            0.3.2      2021-04-29 [1] CRAN (R 4.3.0)
##    esNMA             * 1.1.0001   2024-01-18 [1] bitbucket (triplefin/esNMA@bb41d16)
##    estools           * 0.1.1.0001 2024-01-10 [1] bitbucket (triplefin/estools@03b269b)
##    evaluate            0.23       2023-11-01 [1] CRAN (R 4.3.1)
##    evd                 2.3-6.1    2022-07-04 [1] CRAN (R 4.3.0)
##    fansi               1.0.6      2023-12-08 [1] CRAN (R 4.3.1)
##    farver              2.1.1      2022-07-06 [1] CRAN (R 4.3.0)
##    fastmap             1.1.1      2023-02-24 [1] CRAN (R 4.3.0)
##    flextable           0.9.4      2023-10-22 [1] CRAN (R 4.3.1)
##    fontBitstreamVera   0.1.1      2017-02-01 [1] CRAN (R 4.3.0)
##    fontLiberation      0.1.0      2016-10-15 [1] CRAN (R 4.3.0)
##    fontquiver          0.2.1      2017-02-01 [1] CRAN (R 4.3.0)
##    forcats           * 1.0.0      2023-01-29 [1] CRAN (R 4.3.0)
##    fs                  1.6.3      2023-07-20 [1] CRAN (R 4.3.0)
##    furrr             * 0.3.1      2022-08-15 [1] CRAN (R 4.3.0)
##    future            * 1.33.1     2023-12-22 [1] CRAN (R 4.3.1)
##    gdtools           * 0.3.5      2023-12-09 [1] CRAN (R 4.3.1)
##    generics            0.1.3      2022-07-05 [1] CRAN (R 4.3.0)
##    geomnet             0.3.1      2024-01-18 [1] Github (sctyner/geomnet@030537d)
##    gfonts              0.2.0      2023-01-08 [1] CRAN (R 4.3.0)
##    ggforce             0.4.1      2022-10-04 [1] CRAN (R 4.3.0)
##    ggnewscale          0.4.9      2023-05-25 [1] CRAN (R 4.3.0)
##    ggplot2           * 3.4.4      2023-10-12 [1] CRAN (R 4.3.1)
##    ggraph              2.1.0      2022-10-09 [1] CRAN (R 4.3.0)
##    ggrepel             0.9.5      2024-01-10 [1] CRAN (R 4.3.1)
##    globals             0.16.2     2022-11-21 [1] CRAN (R 4.3.0)
##    glue              * 1.6.2      2022-02-24 [1] CRAN (R 4.3.0)
##    graphlayouts        1.0.2      2023-11-03 [1] CRAN (R 4.3.1)
```

```
##    gridExtra            2.3        2017-09-09 [1] CRAN (R 4.3.0)
##    gt                 * 0.10.1     2024-01-17 [1] CRAN (R 4.3.1)
##    gtable               0.3.4      2023-08-21 [1] CRAN (R 4.3.0)
##    gtsummary          * 1.7.2      2023-07-15 [1] CRAN (R 4.3.0)
##    here                 1.0.1      2020-12-13 [1] CRAN (R 4.3.0)
##    highcharter          0.9.4      2022-01-03 [1] CRAN (R 4.3.0)
##    highr                0.10       2022-12-22 [1] CRAN (R 4.3.0)
##    hms                  1.1.3      2023-03-21 [1] CRAN (R 4.3.0)
##    htmltools            0.5.7      2023-11-03 [1] CRAN (R 4.3.1)
##    htmlwidgets          1.6.4      2023-12-06 [1] CRAN (R 4.3.1)
##    httpcode             0.3.0      2020-04-10 [1] CRAN (R 4.3.0)
##    httpuv               1.6.13     2023-12-06 [1] CRAN (R 4.3.1)
##    httr                 1.4.7      2023-08-15 [1] CRAN (R 4.3.0)
##    igraph               1.6.0      2023-12-11 [1] CRAN (R 4.3.1)
##    inline               0.3.19     2021-05-31 [1] CRAN (R 4.3.0)
##    insight              0.19.7     2023-11-26 [1] CRAN (R 4.3.1)
##    jsonlite             1.8.8      2023-12-04 [1] CRAN (R 4.3.1)
##    knitr                1.45       2023-10-30 [1] CRAN (R 4.3.1)
##    labeling             0.4.3      2023-08-29 [1] CRAN (R 4.3.0)
##    later                1.3.2      2023-12-06 [1] CRAN (R 4.3.1)
##    lattice              0.22-5     2023-10-24 [1] CRAN (R 4.3.2)
##    lazyeval             0.2.2      2019-03-15 [1] CRAN (R 4.3.0)
##    lifecycle            1.0.4      2023-11-07 [1] CRAN (R 4.3.1)
##    listenv              0.9.0      2022-12-16 [1] CRAN (R 4.3.0)
##    lme4                 1.1-35.1   2023-11-05 [1] CRAN (R 4.3.1)
##    loo                  2.6.0      2023-03-31 [1] CRAN (R 4.3.0)
##    lubridate          * 1.9.3      2023-09-27 [1] CRAN (R 4.3.1)
##    magic                1.6-1      2022-11-16 [1] CRAN (R 4.3.0)
##    magick               2.8.2      2023-12-20 [1] CRAN (R 4.3.1)
##    magrittr           * 2.0.3      2022-03-30 [1] CRAN (R 4.3.0)
##    MASS                 7.3-60     2023-05-04 [1] CRAN (R 4.3.2)
##    mathjaxr             1.6-0      2022-02-28 [1] CRAN (R 4.3.0)
##    Matrix               1.6-4      2023-11-30 [1] CRAN (R 4.3.2)
##    matrixStats          1.2.0      2023-12-11 [1] CRAN (R 4.3.1)
##    memoise              2.0.1      2021-11-26 [1] CRAN (R 4.3.0)
##    meta               * 7.0-0      2024-01-12 [1] CRAN (R 4.3.1)
```

```
##    metadat          * 1.2-0      2022-04-06 [1] CRAN (R 4.3.0)
##    metafor            4.4-0      2023-09-27 [1] CRAN (R 4.3.1)
##    mime               0.12       2021-09-28 [1] CRAN (R 4.3.0)
##    miniUI             0.1.1.1    2018-05-18 [1] CRAN (R 4.3.0)
##    minqa              1.2.6      2023-09-11 [1] CRAN (R 4.3.0)
##    multinma         * 0.5.1      2023-05-24 [1] CRAN (R 4.3.0)
##    munsell            0.5.0      2018-06-12 [1] CRAN (R 4.3.0)
##  R NeoEHMnma        * 0.0.0.9000 <NA>       [?] <NA>
##    netmeta          * 2.9-0      2024-01-11 [1] CRAN (R 4.3.1)
##    network            1.18.2     2023-12-05 [1] CRAN (R 4.3.1)
##    nlme               3.1-164    2023-11-27 [1] CRAN (R 4.3.2)
##    nloptr             2.0.3      2022-05-26 [1] CRAN (R 4.3.0)
##    numDeriv           2016.8-1.1 2019-06-06 [1] CRAN (R 4.3.0)
##    officer          * 0.6.3      2023-10-22 [1] CRAN (R 4.3.1)
##    openssl            2.1.1      2023-09-25 [1] CRAN (R 4.3.1)
##    pander             0.6.5      2022-03-18 [1] CRAN (R 4.3.0)
##    parallelly         1.36.0     2023-05-26 [1] CRAN (R 4.3.0)
##    patchwork        * 1.2.0      2024-01-08 [1] CRAN (R 4.3.1)
##    pillar             1.9.0      2023-03-22 [1] CRAN (R 4.3.0)
##    pkgbuild           1.4.3      2023-12-10 [1] CRAN (R 4.3.1)
##    pkgconfig          2.0.3      2019-09-22 [1] CRAN (R 4.3.0)
##    pkgload            1.3.3      2023-09-22 [1] CRAN (R 4.3.1)
##    plotly             4.10.3     2023-10-21 [1] CRAN (R 4.3.1)
##    plyr               1.8.9      2023-10-02 [1] CRAN (R 4.3.1)
##    polyclip           1.10-6     2023-09-27 [1] CRAN (R 4.3.1)
##    profvis            0.3.8      2023-05-02 [1] CRAN (R 4.3.0)
##    promises           1.2.1      2023-08-10 [1] CRAN (R 4.3.0)
##    pryr               0.1.6      2023-01-17 [1] CRAN (R 4.3.0)
##    purrr            * 1.0.2      2023-08-10 [1] CRAN (R 4.3.0)
##    quantmod           0.4.25     2023-08-22 [1] CRAN (R 4.3.0)
##    QuickJSR           1.0.9      2023-12-18 [1] CRAN (R 4.3.1)
##    R.methodsS3        1.8.2      2022-06-13 [1] CRAN (R 4.3.0)
##    R.oo               1.25.0     2022-06-12 [1] CRAN (R 4.3.0)
##    R.utils            2.12.3     2023-11-18 [1] CRAN (R 4.3.1)
##    R6                 2.5.1      2021-08-19 [1] CRAN (R 4.3.0)
##    ragg               1.2.7      2023-12-11 [1] CRAN (R 4.3.1)
```

```
##    rapportools        1.1        2022-03-22 [1] CRAN (R 4.3.0)
##    rbibutils          2.2.16     2023-10-25 [1] CRAN (R 4.3.1)
##    RColorBrewer       1.1-3      2022-04-03 [1] CRAN (R 4.3.0)
##    Rcpp               1.0.11     2023-07-06 [1] CRAN (R 4.3.0)
##    RcppParallel       5.1.7      2023-02-27 [1] CRAN (R 4.3.0)
##    Rdpack             2.6        2023-11-08 [1] CRAN (R 4.3.1)
##    readr            * 2.1.4      2023-02-10 [1] CRAN (R 4.3.0)
##    readxl           * 1.4.3      2023-07-06 [1] CRAN (R 4.3.0)
##    rematch2           2.1.2      2020-05-01 [1] CRAN (R 4.3.0)
##    remotes            2.4.2.1    2023-07-18 [1] CRAN (R 4.3.0)
##    reshape2           1.4.4      2020-04-09 [1] CRAN (R 4.3.0)
##    rio                1.0.1      2023-09-19 [1] CRAN (R 4.3.1)
##    rjags              4-15       2023-11-30 [1] CRAN (R 4.3.1)
##    rlang              1.1.2      2023-11-04 [1] CRAN (R 4.3.1)
##    rlist              0.4.6.2    2021-09-03 [1] CRAN (R 4.3.0)
##    rmarkdown          2.25       2023-09-18 [1] CRAN (R 4.3.1)
##    rprojroot          2.0.4      2023-11-05 [1] CRAN (R 4.3.1)
##    rstan              2.32.5     2024-01-10 [1] CRAN (R 4.3.1)
##    rstantools         2.3.1.1    2023-07-18 [1] CRAN (R 4.3.0)
##    rstudioapi         0.15.0     2023-07-07 [1] CRAN (R 4.3.0)
##    runjags            2.2.2-1.1  2023-08-21 [1] CRAN (R 4.3.0)
##    rvg              * 0.3.3      2023-05-10 [1] CRAN (R 4.3.0)
##    scales             1.3.0      2023-11-28 [1] CRAN (R 4.3.1)
##    sessioninfo        1.2.2      2021-12-06 [1] CRAN (R 4.3.0)
##    shiny              1.8.0      2023-11-17 [1] CRAN (R 4.3.1)
##    sjlabelled       * 1.2.0      2022-04-10 [1] CRAN (R 4.3.0)
##    sna                2.7-2      2023-12-06 [1] CRAN (R 4.3.1)
##    StanHeaders        2.32.5     2024-01-10 [1] CRAN (R 4.3.1)
##    statnet.common     4.9.0      2023-05-24 [1] CRAN (R 4.3.0)
##    stringi            1.8.3      2023-12-11 [1] CRAN (R 4.3.1)
##    stringr          * 1.5.1      2023-11-14 [1] CRAN (R 4.3.1)
##    summarytools     * 1.0.1      2022-05-20 [1] CRAN (R 4.3.0)
##    systemfonts        1.0.5      2023-10-09 [1] CRAN (R 4.3.1)
##    testthat         * 3.2.1      2023-12-02 [1] CRAN (R 4.3.1)
##    textshaping        0.3.7      2023-10-09 [1] CRAN (R 4.3.1)
##    tibble           * 3.2.1      2023-03-20 [1] CRAN (R 4.3.0)
```

```
##    tidygraph          1.3.0     2023-12-18 [1] CRAN (R 4.3.1)
##    tidyr            * 1.3.0     2023-01-24 [1] CRAN (R 4.3.0)
##    tidyselect         1.2.0     2022-10-10 [1] CRAN (R 4.3.0)
##    tidyverse        * 2.0.0     2023-02-22 [1] CRAN (R 4.3.0)
##    timechange         0.2.0     2023-01-11 [1] CRAN (R 4.3.0)
##    truncdist          1.0-2     2016-08-30 [1] CRAN (R 4.3.0)
##    TTR                0.24.4    2023-11-28 [1] CRAN (R 4.3.1)
##    tweenr             2.0.2     2022-09-06 [1] CRAN (R 4.3.0)
##    tzdb               0.4.0     2023-05-12 [1] CRAN (R 4.3.0)
##    urlchecker         1.0.1     2021-11-30 [1] CRAN (R 4.3.0)
##    usethis            2.2.2     2023-07-06 [1] CRAN (R 4.3.0)
##    utf8               1.2.4     2023-10-22 [1] CRAN (R 4.3.1)
##    uuid               1.1-1     2023-08-17 [1] CRAN (R 4.3.0)
##    V8                 4.4.1     2023-12-04 [1] CRAN (R 4.3.1)
##    vctrs              0.6.5     2023-12-01 [1] CRAN (R 4.3.1)
##    viridis            0.6.4     2023-07-22 [1] CRAN (R 4.3.0)
##    viridisLite        0.4.2     2023-05-02 [1] CRAN (R 4.3.0)
##    visNetwork         2.1.2     2022-09-29 [1] CRAN (R 4.3.0)
##    waldo              0.5.2     2023-11-02 [1] CRAN (R 4.3.1)
##    withr              2.5.2     2023-10-30 [1] CRAN (R 4.3.1)
##    writexl            1.4.2     2023-01-06 [1] CRAN (R 4.3.0)
##    xfun             * 0.41      2023-11-01 [1] CRAN (R 4.3.1)
##    xml2               1.3.6     2023-12-04 [1] CRAN (R 4.3.1)
##    xtable             1.8-4     2019-04-21 [1] CRAN (R 4.3.0)
##    xts                0.13.1    2023-04-16 [1] CRAN (R 4.3.0)
##    yaml               2.3.8     2023-12-11 [1] CRAN (R 4.3.1)
##    zip                2.3.0     2023-04-17 [1] CRAN (R 4.3.0)
##    zoo                1.8-12    2023-04-13 [1] CRAN (R 4.3.0)
##
##  [1] /Library/Frameworks/R.framework/Versions/4.3-arm64/Resources/library
##
##  R ── Package was removed from disk.
##
## ──────────────────────────────────────────────────────────────────
```

S2.0 Results

S2.1 References to trials included in the systematic review[1–59]

1. Soni V, Jain S, Chawla D, Khurana S, Rani S. Supplementation of mother's own milk with term versus preterm donor human milk: a randomized controlled trial. Eur J Pediatr. 2023 Feb;182(2):709–18.
2. Monem S, Ezzeldin Z, Said H, Baris S, Tatawy E, Samir S. Bovine Colostrum Supplementation for Prevention of Necrotizing Enterocolitis and Late-Onset Sepsis in Preterm Infants - ProQuest. ProQuest. 2022;20(12):1385–92.
3. Masoli D, Mena P, Dominguez A, Ramolfo P, Vernal P, Pantoja MA, et al. Growth of Very Low Birth Weight Infants Who Received a Liquid Human Milk Fortifier: A Randomized, Controlled Multicenter Trial. J Pediatr Gastroenterol Nutr. 2022 Mar 1;74(3):424–30.
4. Ahnfeldt AM, Aunsholt L, Hansen BM, Hoest B, Jóhannsdóttir V, Kappel SS, et al. Bovine colostrum as a fortifier to human milk in very preterm infants - A randomized controlled trial (FortiColos). Clin Nutr Edinb Scotl. 2023 May;42(5):773–83.
5. Wang C, Han L yan, Zhang L jia, Wang D hua. [Effect of aggressive nutritional support on preterm infants during hospitalization]. Zhonghua Er Ke Za Zhi Chin J Pediatr. 2011 Oct;49(10):771–5.
6. Moltu SJ, Blakstad EW, Strømmen K, Almaas AN, Nakstad B, Rønnestad A, et al. Enhanced feeding and diminished postnatal growth failure in very-low-birth-weight infants. J Pediatr Gastroenterol Nutr. 2014 Mar;58(3):344–51.
7. Chinnappan A, Sharma A, Agarwal R, Thukral A, Deorari A, Sankar MJ. Fortification of Breast Milk With Preterm Formula Powder vs Human Milk Fortifier in Preterm Neonates: A Randomized Noninferiority Trial. JAMA Pediatr. 2021 Aug 1;175(8):790–6.
8. Quan M, Wang D, Gou L, Sun Z, Ma J, Zhang L, et al. Individualized Human Milk Fortification to Improve the Growth of Hospitalized Preterm Infants. Nutr Clin Pract Off Publ Am Soc Parenter Enter Nutr. 2020 Aug;35(4):680–8.
9. O'Connor DL, Kiss A, Tomlinson C, Bando N, Bayliss A, Campbell DM, et al. Nutrient enrichment of human milk with human and bovine milk-based fortifiers for infants born weighing <1250 g: a randomized clinical trial. Am J Clin Nutr. 2018 Jul 1;108(1):108–16.
10. Willeitner A, Anderson M, Lewis J. Highly Concentrated Preterm Formula as an Alternative to Powdered Human Milk Fortifier: A Randomized Controlled Trial. J Pediatr Gastroenterol Nutr. 2017 Nov;65(5):574.
11. Strømmen K, Haag A, Moltu SJ, Veierød MB, Blakstad EW, Nakstad B, et al. Enhanced nutrient supply to very low birth weight infants is associated with higher blood amino acid concentrations and improved growth. Clin Nutr ESPEN. 2017 Apr;18:16–22.
12. Soares FVM, Abranches AD, Méio MDBB, Gomes SC, Villela LD, Moreira MEL. Differences in energy expenditure in human donor milk versus formula milk in preterm newborns: A crossover study. Nutr Burbank Los Angel Cty Calif. 2019 Oct;66:1–4.
13. Schanler RJ, Groh-Wargo SL, Barrett-Reis B, White RD, Ahmad KA, Oliver J, et al. Improved Outcomes in Preterm Infants Fed a Nonacidified Liquid Human Milk Fortifier: A Prospective Randomized Clinical Trial. J Pediatr. 2018 Nov;202:31-37.e2.

14. Rigo J, Hascoët JM, Billeaud C, Picaud JC, Mosca F, Rubio A, et al. Growth and Nutritional Biomarkers of Preterm Infants Fed a New Powdered Human Milk Fortifier: A Randomized Trial. J Pediatr Gastroenterol Nutr. 2017 Oct;65(4):e83–93.
15. Nandakumar A, Pournami F, Prabhakar J, Nair PMC, Jain N. Exclusive Breast Milk vs. Hybrid Milk Feeding for Preterm Babies-A Randomized Controlled Trial Comparing Time to Full Feeds. J Trop Pediatr. 2020 Feb 1;66(1):38–45.
16. Kashaki M, Mazouri A, Bordbar A, Saboute M, Behnamfar Z, Talebi A. Effect of Protein Supplementation on the Growth of Infants Weighing Less than 1,000 Grams Hospitalized on the Neonatal Intensive Care Unit of Akbar Abadi Hospital in Tehran, Iran (2015-2016). Iran J Neonatol. 2018 Sep 1;9(3):49–56.
17. Kadıoğlu Şimşek G, Alyamaç Dizdar E, Arayıcı S, Canpolat FE, Sarı FN, Uraş N, et al. Comparison of the Effect of Three Different Fortification Methods on Growth of Very Low Birth Weight Infants. Breastfeed Med. 2019 Feb;14(1):63–8.
18. Juhl SM, Ye X, Zhou P, Li Y, Iyore EO, Zhang L, et al. Bovine Colostrum for Preterm Infants in the First Days of Life: A Randomized Controlled Pilot Trial. J Pediatr Gastroenterol Nutr. 2018 Mar;66(3):471–8.
19. Hopperton KE, O'Connor DL, Bando N, Conway AM, Ng DVY, Kiss A, et al. Nutrient Enrichment of Human Milk with Human and Bovine Milk-Based Fortifiers for Infants Born <1250 g: 18-Month Neurodevelopment Follow-Up of a Randomized Clinical Trial. Curr Dev Nutr. 2019 Dec;3(12):nzz129.
20. Gupta V, Rebekah G, Sudhakar Y, Santhanam S, Kumar M, Thomas N. A randomized controlled trial comparing the effect of fortification of human milk with an infant formula powder versus unfortified human milk on the growth of preterm very low birth weight infants. J Matern-Fetal Neonatal Med Off J Eur Assoc Perinat Med Fed Asia Ocean Perinat Soc Int Soc Perinat Obstet. 2020 Aug;33(15):2507–15.
21. Adhisivam B, Kohat D, Tanigasalam V, Bhat V, Plakkal N, Palanivel C. Does fortification of pasteurized donor human milk increase the incidence of necrotizing enterocolitis among preterm neonates? A randomized controlled trial. J Matern-Fetal Neonatal Med Off J Eur Assoc Perinat Med Fed Asia Ocean Perinat Soc Int Soc Perinat Obstet. 2019 Oct;32(19):3232–7.
22. Bhat BA, Gupta B. Effects of human milk fortification on morbidity factors in very low birth weight infants. Ann Saudi Med. 2003;23(1–2):28–31.
23. O'Connor D, Shah P, Francis J, Kiss A, Tomlinson C, Bayliss A, et al. Comparison of a human milk-based to a bovine-based human milk fortifier in infants born <1250 grams: A randomized clinical trial. In: PAS Abstracts 2017 [Internet]. 2017 [cited 2024 Feb 10]. Available from: https://www.xcdsystem.com/pas/program/2017/index.cfm?pgid=156
24. Narayanan I, Prakash K, Murthy NS, Gujral VV. Randomised controlled trial of effect of raw and holder pasteurised human milk and of formula supplements on incidence of neonatal infection. Lancet Lond Engl. 1984 Nov 17;2(8412):1111–3.
25. Wang C, Wang D. Application of human milk fortifier in breast-fed preterm infants. J Clin Pediatr. 2009;27(3):259–63.
26. Thakkar P, Aiyer S, Abhishek T, Ukti U. Effect of fortification of human milk on growth and its tolerability in very low birth weight babies – a randomized control trial. Int J Int Med Res. 2016;3(2):38–41.
27. Wu Y, Zhong X yun, Jiang J, Gong H. [Prospective and controlled study on effect of fortified human milk feeding on infants with extremely and very low birth weight during hospital stay]. Beijing Da Xue Xue Bao. 2016 Feb 18;48(1):143–8.

28. Tyson J, Lasky R, Mize C, Richards C, Blair-Smith N, Whyte R, et al. Growth, metabolic response, and development in very-low-birth-weight infants fed banked human milk or enriched formula. I. Neonatal findings. J Pediatr. 1983;103(1):95–104.
29. Sullivan S, Schanler RJ, Kim JH, Patel AL, Trawöger R, Kiechl-Kohlendorfer U, et al. An exclusively human milk-based diet is associated with a lower rate of necrotizing enterocolitis than a diet of human milk and bovine milk-based products. J Pediatr. 2010 Apr;156(4):562-567.e1.
30. Shah SD, Dereddy N, Jones TL, Dhanireddy R, Talati AJ. Early versus Delayed Human Milk Fortification in Very Low Birth Weight Infants-A Randomized Controlled Trial. J Pediatr. 2016 Jul;174:126-131.e1.
31. Schanler RJ, Lau C, Hurst NM, Smith EO. Randomized trial of donor human milk versus preterm formula as substitutes for mothers' own milk in the feeding of extremely premature infants. Pediatrics. 2005 Aug;116(2):400–6.
32. Reis BB, Hall RT, Schanler RJ, Berseth CL, Chan G, Ernst JA, et al. Enhanced growth of preterm infants fed a new powdered human milk fortifier: A randomized, controlled trial. Pediatrics. 2000 Sep;106(3):581–8.
33. O'Connor DL, Gibbins S, Kiss A, Bando N, Brennan-Donnan J, Ng E, et al. Effect of Supplemental Donor Human Milk Compared With Preterm Formula on Neurodevelopment of Very Low-Birth-Weight Infants at 18 Months: A Randomized Clinical Trial. JAMA. 2016 Nov 8;316(18):1897–905.
34. Narayanan I, Bala S, Prakash K, Verma RK, Gujral VV. PARTIAL SUPPLEMENTATION WITH EXPRESSED BREAST-MILK FOR PREVENTION OF INFECTION IN LOW-BIRTH-WEIGHT INFANTS. The Lancet. 1980 Sep 13;316(8194):561–3.
35. Mukhopadhyay K, Narnag A, Mahajan R. Effect of human milk fortification in appropriate for gestation and small for gestation preterm babies: a randomized controlled trial. Indian Pediatr. 2007 Apr;44(4):286–90.
36. Moya F, Sisk PM, Walsh KR, Berseth CL. A new liquid human milk fortifier and linear growth in preterm infants. Pediatrics. 2012 Oct;130(4):e928-935.
37. Miller J, Makrides M, Gibson RA, McPhee AJ, Stanford TE, Morris S, et al. Effect of increasing protein content of human milk fortifier on growth in preterm infants born at <31 wk gestation: a randomized controlled trial1234. Am J Clin Nutr. 2012 Mar 1;95(3):648–55.
38. Martins EC, Krebs VLJ. Effects of the use of fortified raw maternal milk on very low birth weight infants. J Pediatr (Rio J). 2009;85(2):157–62.
39. Lucas A, Morley R, Cole TJ, Gore SM, Lucas PJ, Crowle P, et al. Early diet in preterm babies and developmental status at 18 months. Lancet Lond Engl. 1990 Jun 23;335(8704):1477–81.
40. Lucas A, Morley R, Cole TJ, Gore SM. A randomised multicentre study of human milk versus formula and later development in preterm infants. Arch Dis Child Fetal Neonatal Ed. 1994 Mar;70(2):F141–6.
41. Lucas A, Fewtrell MS, Morley R, Lucas PJ, Baker BA, Lister G, et al. Randomized outcome trial of human milk fortification and developmental outcome in preterm infants. Am J Clin Nutr. 1996 Aug;64(2):142–51.

42. Khorana M, Jiamsajjamongkhon C. Pilot study on growth parameters and nutritional biochemical markers in very low birth weight preterm infants fed human milk fortified with either human milk fortifier or post discharge formula. J Med Assoc Thail Chotmaihet Thangphaet. 2014 Jun;97 Suppl 6:S164-175.
43. Kim JH, Chan G, Schanler R, Groh-Wargo S, Bloom B, Dimmit R, et al. Growth and Tolerance of Preterm Infants Fed a New Extensively Hydrolyzed Liquid Human Milk Fortifier. J Pediatr Gastroenterol Nutr. 2015 Dec;61(6):665–71.
44. Hair AB, Blanco CL, Hawthorne KM, Moreira AG, Lee ML, Rechtman DJ, et al. O-124 Human Milk Cream Enhances Growth When Supplementing Standard Fortification Of An Exclusive Human Milk-based Diet In Vlbw Infants. Arch Dis Child. 2014 Oct 1;99(Suppl 2):A72–3.
45. Hair AB, Bergner EM, Lee ML, Moreira AG, Hawthorne KM, Rechtman DJ, et al. Premature Infants 750–1,250 g Birth Weight Supplemented with a Novel Human Milk-Derived Cream Are Discharged Sooner. Breastfeed Med. 2016 Apr 1;11(3):133–7.
46. Dogra S, Thakur A, Garg P, Kler N. Effect of Differential Enteral Protein on Growth and Neurodevelopment in Infants <1500 g. J Pediatr Gastroenterol Nutr. 2017;64(5):e126–32.
47. Cristofalo EA, Schanler RJ, Blanco CL, Sullivan S, Trawoeger R, Kiechl-Kohlendorfer U, et al. Randomized trial of exclusive human milk versus preterm formula diets in extremely premature infants. J Pediatr. 2013 Dec;163(6):1592-1595.e1.
48. Corpeleijn WE, de Waard M, Christmann V, van Goudoever JB, Jansen-van der Weide MC, Kooi EMW, et al. Effect of Donor Milk on Severe Infections and Mortality in Very Low-Birth-Weight Infants: The Early Nutrition Study Randomized Clinical Trial. JAMA Pediatr. 2016 Jul 1;170(7):654–61.
49. Cheah F, Tiew W, Raja L, Ismail J. A randomized controlled trial comparing the effects of individualized and standardized fortification of expressed breast milk on the growth of preterm infants in the NICU. J Perinat Med. 2015 Oct 1;43(s1):153–431.
50. Bellagamba MP, Carmenati E, D'Ascenzo R, Malatesta M, Spagnoli C, Biagetti C, et al. One Extra Gram of Protein to Preterm Infants From Birth to 1800 g: A Single-Blinded Randomized Clinical Trial. J Pediatr Gastroenterol Nutr. 2016 Jun;62(6):879–84.
51. Barberi I, Marseglia L, Fiamingo C, Arco A, Pagano G. A new formula for premature infants: Effects on growth and nutritional status. Intensive Care Med. 2013 Jun 1;39(1):1–200.
52. Arslanoglu S, Moro GE, Ziegler EE. Adjustable fortification of human milk fed to preterm infants: does it make a difference? J Perinatol Off J Calif Perinat Assoc. 2006 Oct;26(10):614–21.
53. Alizadeh Taheri P, Sajjadian N, Asgharyan Fargi M, Shariat M. Is early breast milk fortification more effective in preterm infants?: a clinical trial. J Perinat Med. 2017 Nov 27;45(8):953–7.
54. Kanmaz HG, Mutlu B, Canpolat FE, Erdeve O, Oguz SS, Uras N, et al. Human Milk Fortification with Differing Amounts of Fortifier and Its Association with Growth and Metabolic Responses in Preterm Infants. J Hum Lact. 2013 Aug 1;29(3):400–5.
55. Svenningsen NW, Lindroth M, Lindquist B. A comparative study of varying protein intake in low birthweight infant feeding. Acta Paediatr Scand Suppl. 1982;296:28–31.
56. Modanlou HD, Lim MO, Hansen JW, Sickles V. Growth, biochemical status, and mineral metabolism in very-low-birth-weight infants receiving fortified preterm human milk. J Pediatr Gastroenterol Nutr. 1986;5(5):762–7.

57. Lucas A, Morley R, Cole TJ, Gore SM, Davis JA, Bamford MF, et al. Early diet in preterm babies and developmental status in infancy. Arch Dis Child. 1989 Nov;64(11):1570–8.
58. Lucas A, Morley R, Cole TJ. Randomised trial of early diet in preterm babies and later intelligence quotient. BMJ. 1998 Nov 28;317(7171):1481–7.
59. Faerk J, Petersen S, Peitersen B, Michaelsen KF. Diet and Bone Mineral Content at Term in Premature Infants. Pediatr Res. 2000 Jan;47(1):148–148.

S2.2 Study Characteristics

Study name	Treatment Details	Total sample size	Mean Gestational Age	Range of Gestational Age	Mean Birth Weight (g)	Proportion Male (%)	Outcomes captured
Kanmaz 2012	Group 1: 1.2 g of HMF added to 30 ml of HM, 3 g/kg/d of protein<br>Group 2: 1.2 g of HMF added to 25 ml HM, 3.3 g/kg/d of protein<br>Group 3: 1.2 g of HMF added to 20 ml of HM, 3.6 g/kg/d of protein	84	28.7	nd	1092	56	Short term growth, feeding intolerance, biochemical markers
Alizadehtaheri 2016	Group 1: fortification on first day<br>Group 2: fortification started when volume of BF reached 75 ml/kg/d, 4.4 of fortifier dissolved in 100 ml of EBM	72	30.34	nd	1295	54	Growth indices, feeding intolerance, NEC, septicemia
Arslanoglu 2006	Group 1: human milk fortified with HMF 5g/100 ml, 0.8 g of protein per 100 ml<br>Group 2: adjustable fortification based on twice weekly BUN. If the BUN was between 9 and 14 mg/dl (3.2–5.0mmol/l), no adjustment was made. Every time the BUN was <9 mg/dl (<3.2 mmol/l), fortification was increased by one level. If the BUN was >14 mg/dl (>5.0 mmol/l), a decrease in fortification by one level was made.	34	31.6	nd	1397	34	Growth indices, BUN, biochemical markers, feeding volume and tolerance
Barberi 2013	Group 1: fortified human milk, started with 1-2g per 100 ml HM, increased to 4 g per 100 ml in one week<br>Group 2: preterm formula	172	31.7	nd	1587	47	Growth, biochemical markers, feeding tolerance
Bellagamba 2016	Group 1: maximum of 2.5 g/kg/d protein in PN, 3.6 g/kg/d in EN<br>Group 2: maximum of 3.5 g/kg/d in PN, 4.6 g/kg/d in EN	164	27	nd	896	57	Growth during hospital stay, growth at 2 years corrected, Bayley III at 24 mo corrected, BUN, cumulate AA/protein intake
Bhat 2003	Group 1: 1 g fortifier to 100 ml EBM once TFI 140 via enteral route, increased to 4 g<br>Group 2: EBM	100	29.4	nd	1242	nd	Weight, biochemical markers, sepsis, ENC, feeding intolerance
Cheah 2015	Group 1: EBM plus HMF (1 pack/25 ml) and additional whey powder to achieve 4.5g/kg/d of protein<br>Group 2: EBM plus HMF (1 pack/25 ml)	34	29.3	nd	1103	nd	Growth parameters, biochemical markers
Corpeleijn 2016	Group 1: Pasteurized donor milk<br>Group 2: preterm formula	373	28.5	nd	830-1275	53	Serious infection (sepsis or meningitis), NEC, or mortality during first 60 days of life
Cristofalo 2013	Group 1: preterm formula, 20-24 kcal/oz<br>Group 2: pasteurized donor milk plus human- derived fortifier	53	27.6	nd	990	43	Growth, duration of parenteral nutrition, duration of hospital stay, days of mechanical ventilation and oxygen therapy, late onset sepsis, NEC, RoP

Study name	Treatment Details	Total sample size	Mean Gestational Age	Range of Gestational Age	Mean Birth Weight (g)	Proportion Male (%)	Outcomes captured
Dogra 2016	Group 1: fortifier 1g/100 ml EBM<br>Group 2:<br>fortifier 0.4 g/100 ml EBM	120	30.2	nd	1219	61	Mean protein intake, occipitofrontal circumference, weight, neurodevelopmental indices and growth at 12-18 months corrected age, feeding tolerance, culture positive sepsis, NEC, biochemical markers
Hair 2016	Group 1: EBM or pasteurized donor milk with pasteurized human fortifier<br>Group 2: Supplementation with 2.5 kcal/ml HM derived cream when EBM or donor HM below 20 kcal/oz	78	27.7	nd	973	52	Medically or surgically managed PDA, blood culture proven sepsis, NEC, BPD, mortality, LOS, PMA at discharge, growth
Hair 2014	Group 1: EBM and pasteurized donor milk, fortified with pasteurized donor HM derived formula once 100 ml/kg/d of enteral feeds if not sooner<br>Group 2: Additional supplementation with human derived cream supplement (2.5 kcal/ml) if HM found to be less than <20 kcal/oz with infrared milk analyzer	78	27.7	nd	971	52	Growth, amount of donor HM derived fortifier used
Kim 2015	Group 1: concentrated liquid HMF containing exclusively hydrolyzed protein, for every 25 ml of HMF, 5 ml of concentrate<br>Group 2: conventional powdered intact protein HMF, for every 25 ml of HM, 1 single packet of fortifier	129	28.8	nd	1175	55	Growth, feeding tolerance, biochemical markers, enteral intake, NEC, sepsis
Khorana 2014	Group 1: HMF once enteral feeds at 100 cc/kg/d, 22-30 cal/oz based on growth<br>Group 2:post discharge formula fortified group once feeds at 100 cc/kg/d, 22-30 cal/oz based on growth	33	30.3	nd	1183	61	Growth, biochemical markers, feeding tolerance
Miller 2012	Group 1: maternal breast milk fortified with HMF 1.4 g protein/100 mL<br>Group 2:<br>Maternal breat milk fortified with HMF 1.0 g protein/100 mL	92	27.8	nd	1034	43	Growth, biochemical markers, feeding tolerance
Moya 2012	Group 1: EBM and/or DBM plus powder HMF<br>Group 2: EBM and/or DMB plus liquid HMF (20% more protein)	146	27.8	nd	1001	47	Growth, biochemical markers, sepsis, NEC, feeding tolerance
Mukhopadhyay 2007	Group 1: EBM + commercial HMF<br>Group 2: EBM + vitamin/mineral supplementation	166	31.1	nd	1230	nd	Weight, biochemical markers, feeding tolerance, length of hospital stay, NEC, sepsis, PDA, BPD, IVH
OConnor 2016	Group 1: mother's own milk plus donor milk with powdered bovine based fortifier and additional protein<br>Group 2:<br>Maternal milk plus preterm formula (3.0 g protein/100 kcal)	363	27.7	nd	996	54	Bayley III at 18 months, mortality and morbidity index, growth
Reis 2000	Group 1: maternal milk or donor milk plus new powdered HMF<br>Group 2: maternal milk or donor milk plus commercially available powdered HMF	119	29.6	nd	1261	51	Growth, biochemical marker, feeding tolerance, sepsis, NEC, respiratory status, steroid/antibiotic therapy
Sullivan 2010	Group 1: maternal milk, donor milk, human milk based HMF when enteral intake reached 100 ml/kg/d<br>Group 2: maternal milk, donor milk, human milk based HMF when enteral intake reached 40 ml/kg/d<br>Group 3: maternal milk, preterm formula, bovine milk based HMF when enteral intake reached 100 ml/kg/d	207	27.2	nd	925	45	Duration of parenteral nutrition, growth, BPD, sepsis, NEC, feeding tolerance
Tyson 1983	Group 1: frozen banked human milk<br>Group 2: preterm formula	76	31.5	nd	1232	39	Growth, biochemical markers, Brazelton Neonatal Behavioural Assessment Scale

Study name	Treatment Details	Total sample size	Mean Gestational Age	Range of Gestational Age	Mean Birth Weight (g)	Proportion Male (%)	Outcomes captured
Thakkar 2016	Group 1: maternal milk plus HMF when enteral feeds 100 ml/kg/d<br>Group 2: maternal milk plus supplements (vit A, D E, folic acid, B complex, Ca, Phos)	61	34.8	nd	1376	44	Growth, feeding tolerance
Rigo 2017	Group 1: new powdered HM fortifier with a higher protein:energy ratio, higher electrolyte and vitamin levels (3.6 g protein/100 kcal)<br>Group 2: standard powdered fortifier (3.10 g protein/100 kcal)	153	28.8	nd	1152	56	Growth, feeding tolerance, adverse events, time to full fortification/enteral feeds, biochemical markers
Schanler 2018	Group 1: human milk plus acidified liquid human milk fortifier<br>Group 2: human milk plus nonacidified liquid human milk fortifier	164	29	nd	1198	45.5	Growth, feeding tolerance, morbidity, biochemical measurements, duration of parenteral nutrition, medication and dietary supplement use, adverse events
Nandakumar 2019	Group 1: mothers milk plus enteral nutrition as needed<br>Group 2: mothers milk plus preterm formula as needed	126	29.4	nd	1150	46.2	Time to reach full enteral feeds, time to regain birth weight, duration of PN, feed intolerance, NEC stage 2 or more, mortality, growth, infections, exclusive breast milk feeding rates at discharge, ROP requiring laser, abnormal cranial US, oxygen dependency at 28 days
Simsek 2019	Group 1: human milk plus bovine fortifier (2.3 g protein/100 ml)<br>Group 2: human milk plus bovine fortifier adjusted based on BUN<br>Group 3: human milk plus bovine fortifier based on breast milk analysis with target of 3.5-4.5 g protein/day	60	29.3	nd	1050	47	Growth, feeding tolerance, frequency of defecation, vomiting, biochemical measurements
Gupta 2018	Group 1: human milk, infant formula powder for fortification, calcium, phosphate, iron, multivitamin drops<br>Group 2: unfortified human milk, calcium, phosphate, iron, multivitamin drops	163	31.2	nd	1239	48	Growth, biochemical parameters, feeding intolerance, sepsis, NEC $\geq$ stage 2
Adhisivam 2019	Group 1: pasteurized donor human milk, bovine powdered milk fortifier, calcium, iron, vitamin E<br>Group 2: pasteurized donor human milk, calcium, iron, vitamin E	80	32.2	nd	1400	nd	NEC, sepsis, mortality, duration of hospital stay, number of days to full enteral feeds, growth
Quan 2019	Group 1: individual fortification based on breast milk assessment of protein, BUN, weight, 5 different levels<br>Group 2: 4 packs HMF per 100 mL huma milk (1.1 g/100 ml)	51	30.4	nd	1376	43	Growth, protein intake, hospital stay duration, feeding tolerance
OConnor 2018	Group 1: maternal milk, donor milk, multivitamin (A, D, C), human derived HMF<br>Group 2: maternal milk, donor milk, multivitamin (A, D, C), bovine derived HMF, protein module	127	27.7	nd	888	43	Interruption in enteral feeding, feeding tolerance, mortality and morbidity index, fecal calprotectin, growth, NEC, sepsis, BPD, ROP
Willeitner 2017	Group 1: maternal milk, powdered HMF<br>Group 2: maternal milk, fortified with liquid preterm formula	70	29	nd	1100	41	Growth, feeding tolerance, death, NEC, sepsis
Juhl 2017	Group 1: maternal milk, bovine colostrum<br>Group 2: maternal milk, donor milk<br>Group 3:<br>Maternal milk, preterm infant formula	40	30.5	nd	1482	58	Feeding tolerance, growth, sepsis, meningitis, BPD, ROP, IVH, PVL, biochemical markers, amino acid analysis, sugar uptake test
Kashaki 2018	Group 1: maternal milk, HMF, protein supplement (0.6-0.8 g/kg/d) when enteral intake 100 ml/kg/d<br>Group 2: maternal milk, HMF	64	29.0	nd	887	nd	Growth, NEC, biochemical markers
Hopperton 2019	Group 1: maternal milk, donor milk, vit A, C, D, human milk based fortifier	109	27.8	nd	897	42	Bayley III, death

Study name	Treatment Details	Total sample size	Mean Gestational Age	Range of Gestational Age	Mean Birth Weight (g)	Proportion Male (%)	Outcomes captured
	Group 2: maternal milk, donor milk with added protein, when enteral intake 100 ml/kg/d, vit A, C, D, bovine milk based fortifier						
Strommen 2017	Group 1: human milk, increased supply of energy, amino acids, long chain polyunsaturated fatty acids, vitamin A (139 kcal/kg/day and 4.0 g./kg/day of AA median during first 4 weeks)<br>Group 2: human milk (124 kcal/kg/day of energy, 3.2 g/kg/day of AA)	44	28.2	nd	1017	64	Growth, amino acid analysis,
Moltu 2013	Group 1: maternal milk, donor milk, HMF (3.6 g/kg/d protein)<br>Group 2: maternal milk, donor milk, HMF, amino acids, polyunsaturated fatty acids, vitamin A (4.4 g/kg/d protein, 10% more energy, 20% more protein)	44	28.3	nd	1017	64	Growth, NEC, BPD, RoP, PDA, sepsis, IVH
Schanler 2005	Group 1: maternal milk, donor milk<br>Group 2: maternal milk, preterm formula<br>Group 3: maternal milk only	243	27	nd	968	52	NEC, meningitis, sepsis, UTI, growth, skin to skin contact, duration of hospital stay
Faerk 2000	Group 1: maternal milk, phosphate (10 mg/100 ml)<br>Group 2: maternal milk, fortifier with phosphate and calcium (0.4 g protein, 35 mg Ca, 17 mg phos per 100 mL)<br>Group 3: maternal milk, preterm formula	127					Bone mineral content, growth
Narayanan 1980	Group 1: Expressed milk in day, formula at night<br>Group 2: formula	70	nd	28 weeks->37 weeks	1001-2500 g	49	Infection
Narayanan 1984	Group 1: raw expressed human milk<br>Group 2: pasteurized human milk<br>Group 3: raw human milk in day, formula at night<br>Group 4: pasteurized human milk in day, formula at night	226	nd	28->37 weeks	1001-2500 g	53	Infection
Modanlou 1986	Group 1: mothers own milk<br>Group 2: fortified mothers own milk<br>Group 3: preterm formula	30	nd	nd	1175	nd	Biochemical markers, bone density, feeding tolerance, growth
Svenningsen 1982	Group 1: human milk with 1.6 g protein per 100 kcal<br>Group 2: formula with 2.3 g protein per 100 kcal<br>Group 3: formula with 3.0 g protein per 100 kcal	48	31	nd	1405	nd	Growth, biochemical markers, neurodevelopmental examinations at 2 years
Wang 2012	Group 1: preterm formula alone<br>Group 2: mothers own milk, preterm formula, bovine fortifier	125	nd	nd	1472	nd	Growth, enteral intake, parenteral intake
Martins 2009	Group 1: human milk<br>Group 2: fortified human milk	40	nd	≤34 weeks	1208	58	Growth, infection, feeding tolerance
Shah 2016	Group 1: human milk with acidified liquid HMF beginning at 20 mL/kg/d<br>Group 2: human milk with acidified liquid HMF beginning at 100 mL/kg/d	100	27.5 (26,30)<br>28 (26, 30)<br>n=50 n=50	nd	990 (780, 1200)<br>990 (840, 1250)<br>N=50<br>n=50	55	Number of days to full enteral feeds
Wu 2016	Group 1: human milk with human milk fortification<br>Group 2: premature formula alone	122	29.8	nd	1278.36	44	Growth, duration of hospital stay, biochemical markers, bone density, complications

Study name	Treatment Details	Total sample size	Mean Gestational Age	Range of Gestational Age	Mean Birth Weight (g)	Proportion Male (%)	Outcomes captured
O'Connor 2017		127					
Lucas 1994a	Group 1: human donor milk<br>Group 2: preterm formula	159					
Lucas 1994b	Group 1: human donor milk<br>Group 2: human milk with a preterm formula	343					
Lucas 1998a	Group 1: term formula<br>Group 2: preterm formula	160					
Lucas 1998b	Group 1: term formula<br>Group 2: human milk with preterm formula	264					
Lucas 1989a	Group 1: human donor milk<br>Group 2: preterm formula	159					
Lucas 1989b	Group 1: human donor milk<br>Group 2: human milk with a preterm formula	343					
Lucas 1990a	Group 1: term formula<br>Group 2: preterm formula	926					
Lucas 1990b	Group 1: term formula<br>Group 2: human milk with preterm formula	264					
Lucas 1996	Group 1: human milk with phosphate and vitamins<br>Group 2: human milk	275					
Soares 2019		29					
Chinnappan 2021		122					
Masoli 2021	Group 1: human milk with a liquid human milk fortifier<br>Group 2: human milk with a powder fortifier	158	27.7 (1.9) and 27.9 (1.7) n=76 and n=82)	nd	1035.2 (225.1) and 1031.9 (178.1) n=76 and n=82	48	Growth, NEC, BPD, sepsis, full enteral feed
Wang 2009	Group 1: human milk<br>Group 2: preterm formula alone	24	30.6 (2.9) and 31.6 (1.9) n=11 and n=13	nd	1280 (286) and 1436 (201) n=11 and n=13	42	Growth, full enteral feel
Soni 2022	Group 1: preterm human milk<br>Group 2: pasteurized term human milk	102	31.3 (2.8) and 31.8 (2.4) n=54 and n=48	nd	1262 (184) and 1253 (181) n=54 and n=48	60	Growth, full enteral feed, NEC, feed intolerance
Monem 2022	Group 1: powdered bovine colostrum<br>Group 2: preterm formula alone	120	32.17 ± 1.564 and	nd	1.6363 ± .37975 and	57	Full enteral feed, mortality, NEC, sepsis, feed intolerance

Study name	Treatment Details	Total sample size	Mean Gestational Age	Range of Gestational Age	Mean Birth Weight (g)	Proportion Male (%)	Outcomes captured
			31.88 ± 1.878 (n=60 and n=60)		1.6209 ± .34139 (n=60 and n=60)		
Ahnfeldt 2023	Group 1: powdered bovine colostrum<br>Group 2: bovine milk-based fortifier	232	28+5 (26-30, n=117) and 28+6 (26-30, n=115)	nd	1164 ± 323 (n=117) 1170 ± 333 (n=115)	43	Growth, Sepsis, NEC, BPD, ROP, IVH

S2.3 Risk of bias assessment

Study	registered or published protocol	Random sequence generation (selection bias)	Allocation concealment (selection bias)	Blinding of participants (performance bias)	Blinding of personnel (performance bias)	Blinding of outcome assessor (detection bias)	Incomplete outcome data (attrition bias)	Selective Reporting (reporting bias)	Additional Bias	Overall
wang 2012										
kanmaz 2012										
alizadehtaheri 2016										
arslanoglu 2006										
barberi 2013										
bellagamba 2016										
bhat 2003										
cheah 2015										
corpeleijn 2016										
cristofalo 2013										
dogra 2016										
hair 2016										
hair 2014										
kim 2015										
khorana 2014										
lucas 1990a										

lucas 1996										
lucas 1994a										
lucas 1990b										
martins 2009										
miller 2012										
moya 2012										
mukhopadhyay 2007										
narayanan 1980										
oconnor 2016										
reis 2000										
shah 2016										
sullivan 2010										
tyson 1983										
wu 2016										
thakkar 2016										
wang 2009										
narayanan 1984										
oconnor 2017										
svenningsen 1982										
lucas 1994b										
lucas 1998a										
lucas 1998b										
lucas 1989a										
lucas 1989b										
modanlou 1986										
faerk 2000										
rigo 2017										
schanler 2018										

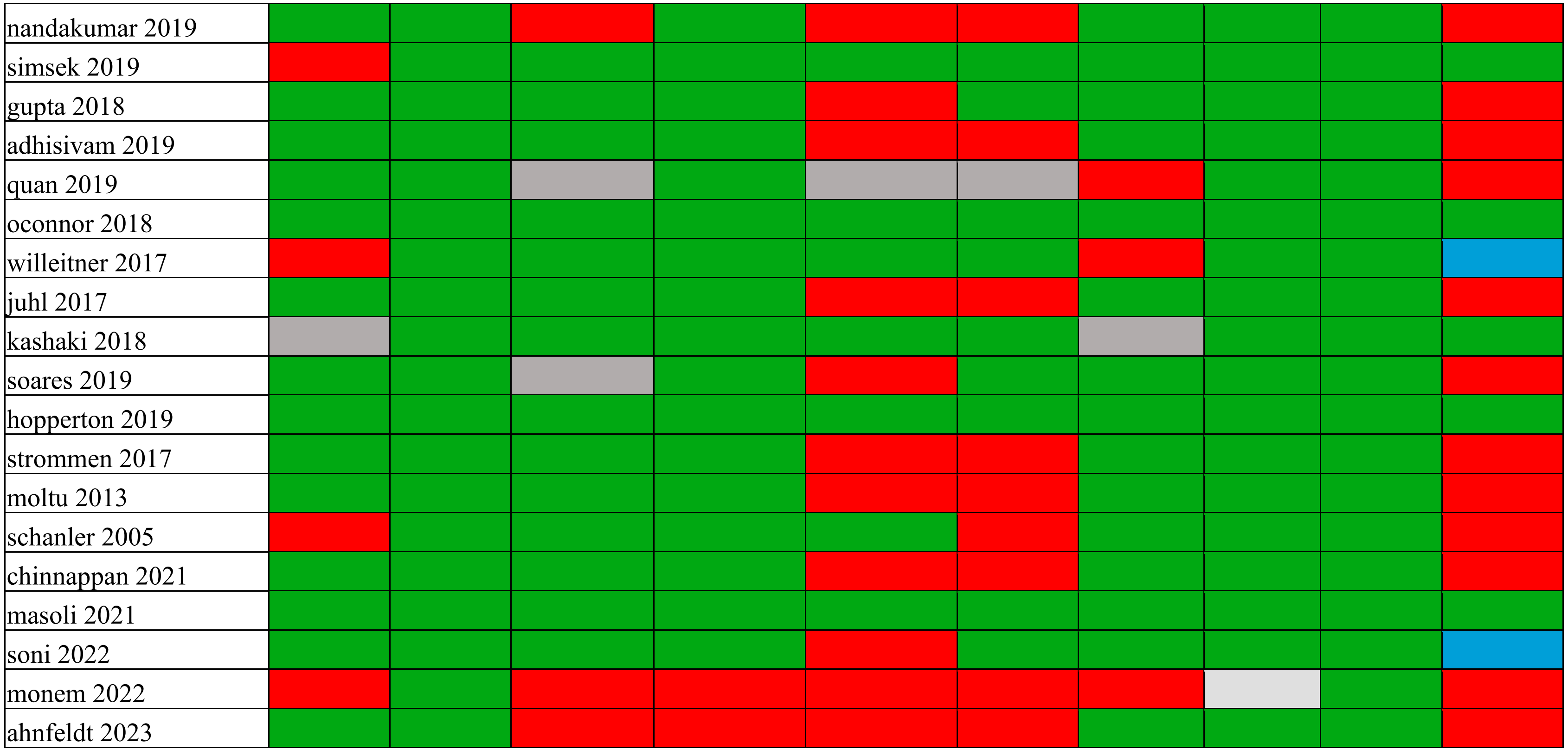

*green=low risk, red=high risk, grey=NA, yellow=medium risk

S2.4 League tables

Mortality

Fixed effects model for interventions

BIN - Unadjusted NMA (Odds Ratio)

mom+dm+hdf																				
0.92 (0.29 to 3.83)	mom+dm+ef																			
0.89 (0.28 to 3.36)	1 (0.32 to 2.48)	mom+pdm+pbov																		
0.86 (0.26 to 3.62)	0.98 (0.3 to 2.59)	0.98 (0.36 to 2.74)	mom+dm+hp+pbov																	
0.83 (0.25 to 3.38)	0.96 (0.28 to 2.44)	0.97 (0.33 to 2.54)	0.99 (0.31 to 2.8)	mom+dm+hpfa+lf																
0.83 (0.23 to 3.68)	0.96 (0.27 to 2.68)	0.97 (0.32 to 2.74)	0.99 (0.31 to 3.01)	1 (0.34 to 3.12)	mom+dm+ehlf															
0.76 (0.21 to 2.71)	0.91 (0.21 to 1.99)	0.91 (0.26 to 2)	0.94 (0.25 to 2.27)	0.96 (0.26 to 2.32)	0.95 (0.25 to 2.43)	mom+dm+lf+pbov														
0.74 (0.19 to 2.99)	0.89 (0.2 to 2.16)	0.91 (0.24 to 2.16)	0.93 (0.23 to 2.32)	0.95 (0.24 to 2.43)	0.95 (0.24 to 2.42)	1 (0.31 to 2.95)	mom+dm+lbov													
0.65 (0.22 to 1.91)	0.73 (0.19 to 1.89)	0.74 (0.23 to 1.92)	0.77 (0.21 to 2.1)	0.79 (0.23 to 2.14)	0.79 (0.2 to 2.24)	0.87 (0.27 to 2.51)	0.89 (0.26 to 2.75)	mom+dm												
0.62 (0.21 to 1.76)	0.69 (0.18 to 1.75)	0.7 (0.23 to 1.75)	0.73 (0.2 to 1.88)	0.75 (0.22 to 1.92)	0.75 (0.19 to 2.07)	0.82 (0.27 to 2.25)	0.83 (0.26 to 2.49)	0.94 (0.65 to 1.4)	mom+pform											
0.6 (0.16 to 2.41)	0.68 (0.15 to 2.11)	0.69 (0.17 to 2.32)	0.71 (0.16 to 2.54)	0.73 (0.17 to 2.6)	0.73 (0.16 to 2.71)	0.79 (0.22 to 3.26)	0.8 (0.2 to 3.73)	0.89 (0.34 to 3.09)	0.94 (0.38 to 3.13)	mom+pf+lbov										
0.6 (0.21 to 1.62)	0.71 (0.18 to 1.28)	0.71 (0.25 to 1.22)	0.75 (0.21 to 1.39)	0.77 (0.23 to 1.43)	0.77 (0.2 to 1.57)	0.84 (0.29 to 1.7)	0.86 (0.27 to 1.99)	0.91 (0.43 to 1.99)	0.96 (0.48 to 1.95)	1.04 (0.28 to 2.56)	mom+dm+pbov									
0.58 (0.18 to 1.7)	0.66 (0.15 to 1.7)	0.66 (0.19 to 1.73)	0.69 (0.17 to 1.84)	0.71 (0.18 to 1.89)	0.71 (0.16 to 1.99)	0.78 (0.23 to 2.19)	0.79 (0.21 to 2.39)	0.9 (0.43 to 1.71)	0.97 (0.5 to 1.63)	1.01 (0.26 to 2.54)	0.97 (0.45 to 2.07)	mom+pf+pdf								
0.55 (0.13 to 2.13)	0.63 (0.11 to 1.88)	0.64 (0.13 to 1.98)	0.66 (0.13 to 2.22)	0.68 (0.14 to 2.26)	0.68 (0.12 to 2.44)	0.73 (0.17 to 2.8)	0.74 (0.15 to 3.09)	0.82 (0.26 to 2.58)	0.86 (0.29 to 2.65)	0.96 (0.25 to 2.48)	0.88 (0.31 to 2.95)	0.92 (0.31 to 3.06)	mom+pf+hp+pbov							
0.52 (0.11 to 2.32)	0.56 (0.11 to 2.48)	0.58 (0.13 to 2.49)	0.6 (0.11 to 2.64)	0.62 (0.12 to 2.73)	0.62 (0.11 to 2.81)	0.69 (0.15 to 3.12)	0.7 (0.14 to 3.32)	0.79 (0.25 to 2.52)	0.83 (0.29 to 2.51)	0.87 (0.18 to 3.64)	0.87 (0.24 to 3.22)	0.9 (0.27 to 3.08)	0.97 (0.21 to 4.44)	mom						
0.53 (0.18 to 1.4)	0.6 (0.16 to 1.34)	0.61 (0.2 to 1.36)	0.64 (0.18 to 1.46)	0.65 (0.19 to 1.53)	0.65 (0.17 to 1.63)	0.71 (0.24 to 1.81)	0.72 (0.22 to 1.99)	0.81 (0.42 to 1.57)	0.85 (0.48 to 1.52)	0.94 (0.29 to 1.99)	0.88 (0.51 to 1.52)	0.91 (0.5 to 1.74)	0.99 (0.35 to 2.56)	1.01 (0.29 to 3.39)	mom+pf+pbov					
0.5 (0.12 to 1.77)	0.58 (0.1 to 1.63)	0.59 (0.12 to 1.68)	0.61 (0.11 to 1.81)	0.63 (0.12 to 1.91)	0.63 (0.1 to 2.04)	0.69 (0.15 to 2.32)	0.7 (0.14 to 2.46)	0.77 (0.25 to 2.08)	0.82 (0.27 to 2.1)	0.9 (0.21 to 2.13)	0.83 (0.28 to 2.36)	0.85 (0.28 to 2.49)	0.96 (0.28 to 2.62)	0.95 (0.2 to 4.01)	0.97 (0.34 to 2.38)	mom+bovc				
0.42 (0.1 to 2.27)	0.47 (0.1 to 2)	0.48 (0.11 to 2.07)	0.49 (0.11 to 2.27)	0.51 (0.12 to 2.37)	0.5 (0.11 to 2.43)	0.56 (0.13 to 3.02)	0.56 (0.13 to 3.4)	0.64 (0.21 to 2.84)	0.67 (0.23 to 2.88)	0.7 (0.17 to 3.47)	0.69 (0.22 to 3.34)	0.71 (0.23 to 3.6)	0.75 (0.2 to 4.62)	0.83 (0.17 to 4.76)	0.78 (0.26 to 3.52)	0.81 (0.24 to 5.01)	mom+if			
0.4 (0.1 to 1.7)	0.44 (0.09 to 1.58)	0.45 (0.11 to 1.63)	0.47 (0.1 to 1.76)	0.47 (0.1 to 1.89)	0.47 (0.09 to 1.95)	0.52 (0.13 to 2.3)	0.52 (0.12 to 2.52)	0.59 (0.2 to 2.11)	0.63 (0.22 to 2.12)	0.66 (0.17 to 2.59)	0.65 (0.21 to 2.46)	0.67 (0.23 to 2.57)	0.72 (0.2 to 3.19)	0.76 (0.16 to 3.74)	0.73 (0.27 to 2.61)	0.78 (0.26 to 3.03)	0.97 (0.25 to 2.66)	mom+bovc		
0.41 (0.12 to 1.29)	0.45 (0.1 to 1.37)	0.46 (0.13 to 1.35)	0.48 (0.12 to 1.45)	0.5 (0.12 to 1.52)	0.5 (0.11 to 1.59)	0.55 (0.15 to 1.77)	0.55 (0.14 to 1.93)	0.63 (0.31 to 1.27)	0.66 (0.34 to 1.3)	0.69 (0.18 to 2.12)	0.69 (0.27 to 1.71)	0.71 (0.3 to 1.72)	0.76 (0.22 to 2.71)	0.8 (0.21 to 2.89)	0.78 (0.32 to 1.82)	0.82 (0.26 to 2.93)	0.97 (0.2 to 3.49)	1.04 (0.26 to 3.61)	pform	
0.32 (0.09 to 1.09)	0.36 (0.07 to 1.14)	0.36 (0.09 to 1.12)	0.38 (0.08 to 1.2)	0.39 (0.09 to 1.24)	0.39 (0.08 to 1.26)	0.43 (0.11 to 1.38)	0.43 (0.1 to 1.49)	0.48 (0.19 to 1.23)	0.51 (0.21 to 1.21)	0.54 (0.13 to 1.62)	0.53 (0.2 to 1.35)	0.54 (0.21 to 1.4)	0.59 (0.15 to 1.97)	0.61 (0.15 to 2.38)	0.6 (0.25 to 1.41)	0.64 (0.19 to 2.08)	0.83 (0.18 to 1.7)	0.88 (0.23 to 1.85)	0.78 (0.25 to 2.34)	mom+pf

Random effects model for interventions

BIN - Unadjusted NMA (Odds Ratio)

mom+dm+hdf																				
0.86 (0.26 to 3.66)	mom+dm+ef																			
0.86 (0.25 to 3.31)	0.99 (0.33 to 2.39)	mom+pdm+pbov																		
0.82 (0.22 to 3.39)	0.97 (0.29 to 2.47)	0.98 (0.33 to 2.72)	mom+dm+hp+pbov																	
0.81 (0.22 to 3.7)	0.97 (0.27 to 2.7)	0.98 (0.31 to 2.94)	1 (0.33 to 3.13)	mom+dm+ehlf																
0.79 (0.22 to 3.38)	0.96 (0.28 to 2.39)	0.97 (0.33 to 2.5)	0.99 (0.32 to 2.72)	1 (0.29 to 3.13)	mom+dm+hpfa+lf															
0.73 (0.2 to 2.91)	0.92 (0.22 to 2.04)	0.93 (0.27 to 2.15)	0.96 (0.25 to 2.43)	0.97 (0.23 to 2.56)	0.97 (0.26 to 2.42)	mom+dm+lf+pbov														
0.72 (0.18 to 2.98)	0.91 (0.2 to 2.01)	0.92 (0.24 to 2.13)	0.95 (0.23 to 2.39)	0.95 (0.24 to 2.37)	0.96 (0.25 to 2.43)	0.99 (0.31 to 3.05)	mom+dm+lbov													
0.66 (0.2 to 1.95)	0.76 (0.19 to 2)	0.78 (0.23 to 2.08)	0.81 (0.21 to 2.27)	0.83 (0.18 to 2.36)	0.84 (0.22 to 2.34)	0.89 (0.28 to 2.73)	0.91 (0.25 to 3.03)	mom+dm												
0.61 (0.21 to 1.67)	0.72 (0.19 to 1.72)	0.74 (0.23 to 1.77)	0.77 (0.21 to 1.96)	0.79 (0.17 to 2.1)	0.79 (0.22 to 2.02)	0.83 (0.27 to 2.48)	0.86 (0.24 to 2.62)	0.93 (0.59 to 1.44)	mom+pform											
0.6 (0.15 to 2.39)	0.7 (0.16 to 2.16)	0.72 (0.18 to 2.24)	0.76 (0.18 to 2.46)	0.75 (0.16 to 2.84)	0.76 (0.18 to 2.68)	0.8 (0.22 to 3.35)	0.83 (0.21 to 3.57)	0.9 (0.32 to 3.25)	0.96 (0.36 to 3.23)	mom+pf+lbov										
0.58 (0.18 to 1.71)	0.69 (0.15 to 1.75)	0.71 (0.18 to 1.86)	0.73 (0.18 to 2)	0.75 (0.15 to 2.09)	0.75 (0.18 to 2.1)	0.8 (0.22 to 2.44)	0.82 (0.2 to 2.6)	0.89 (0.4 to 1.83)	0.98 (0.5 to 1.68)	1 (0.27 to 2.64)	mom+pf+pdf									
0.59 (0.2 to 1.63)	0.73 (0.18 to 1.29)	0.74 (0.23 to 1.28)	0.78 (0.22 to 1.42)	0.8 (0.18 to 1.57)	0.8 (0.23 to 1.47)	0.86 (0.27 to 1.73)	0.87 (0.25 to 2.1)	0.91 (0.4 to 1.97)	0.97 (0.46 to 2)	1.02 (0.28 to 2.6)	1.02 (0.44 to 2.29)	mom+dm+pbov								
0.53 (0.11 to 2.43)	0.58 (0.11 to 2.69)	0.61 (0.12 to 2.63)	0.63 (0.13 to 2.83)	0.65 (0.11 to 2.91)	0.65 (0.13 to 2.9)	0.7 (0.15 to 3.19)	0.72 (0.13 to 3.53)	0.81 (0.24 to 2.67)	0.86 (0.28 to 2.64)	0.88 (0.19 to 3.72)	0.91 (0.26 to 3.3)	0.89 (0.24 to 3.4)	mom							
0.55 (0.12 to 2)	0.65 (0.12 to 1.95)	0.67 (0.13 to 2.06)	0.69 (0.13 to 2.23)	0.7 (0.12 to 2.37)	0.71 (0.14 to 2.3)	0.76 (0.16 to 2.83)	0.77 (0.16 to 2.99)	0.82 (0.24 to 2.59)	0.89 (0.29 to 2.55)	0.95 (0.26 to 2.46)	0.92 (0.28 to 2.95)	0.9 (0.31 to 2.98)	1.02 (0.22 to 4.58)	mom+pf+hp+pbov						
0.51 (0.12 to 1.82)	0.61 (0.11 to 1.65)	0.63 (0.13 to 1.71)	0.65 (0.12 to 1.91)	0.66 (0.11 to 2.07)	0.67 (0.12 to 1.98)	0.71 (0.16 to 2.27)	0.73 (0.15 to 2.49)	0.78 (0.24 to 2.18)	0.84 (0.27 to 2.18)	0.92 (0.22 to 2.1)	0.88 (0.27 to 2.5)	0.85 (0.29 to 2.38)	0.96 (0.2 to 4.13)	0.98 (0.29 to 2.58)	mom+bovc					
0.53 (0.17 to 1.44)	0.62 (0.17 to 1.42)	0.64 (0.2 to 1.46)	0.67 (0.18 to 1.56)	0.68 (0.16 to 1.67)	0.68 (0.19 to 1.62)	0.73 (0.23 to 1.84)	0.75 (0.21 to 2.06)	0.81 (0.38 to 1.66)	0.87 (0.46 to 1.58)	0.94 (0.28 to 1.94)	0.91 (0.46 to 1.78)	0.89 (0.49 to 1.65)	1 (0.28 to 3.43)	0.99 (0.35 to 2.67)	1.02 (0.42 to 2.87)	mom+pf+pbov				
0.43 (0.09 to 2.09)	0.49 (0.09 to 1.95)	0.5 (0.11 to 2.12)	0.52 (0.1 to 2.24)	0.52 (0.1 to 2.48)	0.53 (0.11 to 2.42)	0.57 (0.13 to 3.13)	0.57 (0.12 to 3.2)	0.64 (0.19 to 2.74)	0.68 (0.22 to 2.67)	0.7 (0.16 to 3.17)	0.71 (0.21 to 3.37)	0.7 (0.21 to 3.24)	0.79 (0.16 to 4.55)	0.76 (0.19 to 4.43)	0.81 (0.22 to 4.48)	0.79 (0.24 to 3.4)	mom+lf			
0.4 (0.09 to 1.61)	0.46 (0.09 to 1.57)	0.47 (0.1 to 1.62)	0.49 (0.1 to 1.84)	0.5 (0.09 to 1.96)	0.5 (0.1 to 1.9)	0.53 (0.12 to 2.17)	0.54 (0.11 to 2.33)	0.6 (0.19 to 2.02)	0.64 (0.21 to 1.97)	0.66 (0.16 to 2.59)	0.67 (0.2 to 2.52)	0.66 (0.21 to 2.34)	0.74 (0.15 to 4.09)	0.72 (0.2 to 3.04)	0.76 (0.24 to 2.93)	0.75 (0.24 to 2.49)	0.98 (0.24 to 2.71)	mom+bovc		
0.41 (0.11 to 1.4)	0.47 (0.11 to 1.5)	0.49 (0.13 to 1.54)	0.5 (0.12 to 1.66)	0.52 (0.11 to 1.76)	0.52 (0.12 to 1.68)	0.56 (0.15 to 1.94)	0.57 (0.14 to 2.07)	0.63 (0.28 to 1.34)	0.67 (0.31 to 1.38)	0.69 (0.18 to 2.25)	0.71 (0.28 to 1.77)	0.7 (0.26 to 1.93)	0.78 (0.19 to 3.16)	0.77 (0.21 to 2.88)	0.8 (0.25 to 2.92)	0.78 (0.31 to 1.98)	0.97 (0.21 to 3.86)	1.03 (0.27 to 3.89)	pform	
0.31 (0.08 to 1.08)	0.37 (0.07 to 1.21)	0.38 (0.09 to 1.22)	0.39 (0.08 to 1.26)	0.4 (0.07 to 1.34)	0.4 (0.08 to 1.31)	0.43 (0.11 to 1.41)	0.45 (0.1 to 1.52)	0.48 (0.18 to 1.27)	0.51 (0.2 to 1.22)	0.54 (0.13 to 1.61)	0.54 (0.19 to 1.48)	0.53 (0.19 to 1.43)	0.59 (0.14 to 2.48)	0.59 (0.16 to 1.92)	0.63 (0.2 to 1.91)	0.6 (0.23 to 1.46)	0.83 (0.19 to 1.68)	0.88 (0.25 to 1.87)	0.76 (0.24 to 2.37)	mom+pf

Fixed effects model for classes

BIN - Unadjusted NMA (Odds Ratio)

mom+dm+hm							
0.83 (0.25 to 3.26)	mom+dm+bov						
0.68 (0.18 to 2.76)	0.84 (0.24 to 2.36)	mom+dm					
0.66 (0.21 to 2.12)	0.77 (0.32 to 1.73)	0.95 (0.43 to 2.15)	mom+form				
0.57 (0.16 to 2.13)	0.69 (0.24 to 1.67)	0.83 (0.28 to 2.51)	0.87 (0.4 to 1.94)	mom+form+bov			
0.55 (0.11 to 3.08)	0.66 (0.15 to 2.76)	0.8 (0.21 to 3.4)	0.86 (0.27 to 2.85)	0.99 (0.24 to 4.1)	mom		
0.45 (0.11 to 1.97)	0.55 (0.15 to 1.72)	0.65 (0.21 to 2.24)	0.69 (0.28 to 1.81)	0.79 (0.23 to 2.6)	0.81 (0.18 to 3.69)	form	
0.41 (0.1 to 1.78)	0.48 (0.15 to 1.51)	0.58 (0.18 to 2.25)	0.62 (0.25 to 1.7)	0.7 (0.25 to 2.23)	0.72 (0.17 to 3.37)	0.9 (0.25 to 3.61)	mom+bov

Random effects model for classes

BIN - Unadjusted NMA (Odds Ratio)

mom+dm+hm							
0.81 (0.24 to 3.17)	mom+dm+bov						
0.69 (0.17 to 2.82)	0.86 (0.24 to 2.43)	mom+dm					
0.65 (0.2 to 2.05)	0.8 (0.31 to 1.77)	0.94 (0.42 to 2.22)	mom+form				
0.58 (0.15 to 2.11)	0.72 (0.25 to 1.69)	0.84 (0.28 to 2.66)	0.89 (0.4 to 1.97)	mom+form+bov			
0.57 (0.11 to 3.02)	0.69 (0.16 to 2.85)	0.82 (0.19 to 3.51)	0.88 (0.26 to 2.89)	0.98 (0.25 to 4.05)	mom		
0.46 (0.1 to 2.05)	0.56 (0.15 to 1.88)	0.66 (0.2 to 2.33)	0.7 (0.27 to 1.84)	0.79 (0.24 to 2.81)	0.8 (0.17 to 3.9)	form	
0.41 (0.1 to 1.77)	0.5 (0.15 to 1.48)	0.58 (0.17 to 2.24)	0.62 (0.24 to 1.67)	0.7 (0.24 to 2.14)	0.72 (0.15 to 3.36)	0.89 (0.23 to 3.47)	mom+bov

NEC

Fixed effects model for interventions

BIN - Unadjusted NMA (Odds Ratio)

mom+dm+shdf																							
0.94 (0.34 to 1.88)	mom+dm+hdf																						
0.52 (0.19 to 1.66)	0.57 (0.23 to 1.87)	mom+dm+hpfa+lf																					
0.49 (0.16 to 1.44)	0.53 (0.21 to 1.5)	0.97 (0.31 to 2.2)	mom+dm+lf+pbov																				
0.48 (0.16 to 1.44)	0.53 (0.21 to 1.6)	0.96 (0.3 to 2.28)	1 (0.38 to 2.64)	mom+dm+ehlf																			
0.48 (0.17 to 1.24)	0.53 (0.22 to 1.25)	0.96 (0.3 to 2.04)	0.99 (0.37 to 2.32)	1 (0.35 to 2.44)	mom+dm+ef																		
0.48 (0.15 to 1.3)	0.53 (0.2 to 1.3)	0.91 (0.29 to 2.23)	0.98 (0.34 to 2.43)	0.98 (0.33 to 2.5)	1 (0.39 to 2.39)	mom+dm																	
0.47 (0.15 to 1.49)	0.52 (0.2 to 1.62)	0.95 (0.26 to 2.2)	0.99 (0.33 to 2.75)	0.99 (0.33 to 2.83)	0.99 (0.37 to 2.79)	1 (0.38 to 3.04)	mom+pdm+pbov																
0.47 (0.15 to 1.34)	0.51 (0.2 to 1.42)	0.94 (0.27 to 2.07)	0.98 (0.34 to 2.43)	0.98 (0.32 to 2.51)	0.99 (0.38 to 2.41)	0.97 (0.38 to 2.7)	0.99 (0.33 to 2.71)	mom+dm+hp+pbo															
0.46 (0.15 to 1.2)	0.51 (0.21 to 1.16)	0.88 (0.29 to 1.99)	0.95 (0.36 to 2.15)	0.96 (0.33 to 2.23)	0.96 (0.41 to 2.11)	0.97 (0.59 to 1.58)	0.97 (0.33 to 2.39)	0.99 (0.38 to 2.32)	mom+pform														
0.46 (0.14 to 1.28)	0.5 (0.19 to 1.4)	0.93 (0.25 to 2.01)	0.97 (0.31 to 2.34)	0.98 (0.3 to 2.37)	0.98 (0.36 to 2.37)	0.96 (0.36 to 2.65)	0.99 (0.31 to 2.69)	1 (0.35 to 2.58)	0.99 (0.4 to 2.52)	mom+dm+af													
0.44 (0.17 to 0.99)	0.49 (0.23 to 0.97)	0.9 (0.32 to 1.43)	0.95 (0.41 to 1.64)	0.96 (0.39 to 1.69)	0.96 (0.47 to 1.64)	0.93 (0.45 to 1.88)	0.97 (0.36 to 1.87)	0.98 (0.44 to 1.8)	0.97 (0.53 to 1.78)	0.99 (0.45 to 1.94)	mom+dm+pbov												
0.43 (0.11 to 1.16)	0.47 (0.16 to 1.2)	0.88 (0.19 to 1.8)	0.93 (0.24 to 2.06)	0.94 (0.25 to 2.02)	0.94 (0.27 to 2.1)	0.89 (0.29 to 2.34)	0.95 (0.23 to 2.37)	0.95 (0.27 to 2.31)	0.93 (0.31 to 2.24)	0.97 (0.28 to 2.43)	0.98 (0.39 to 1.95)	mom+dm+lbov											
0.42 (0.11 to 1.11)	0.46 (0.16 to 1.16)	0.88 (0.19 to 1.72)	0.92 (0.23 to 1.97)	0.93 (0.23 to 1.98)	0.93 (0.28 to 2)	0.88 (0.29 to 2.3)	0.94 (0.23 to 2.13)	0.95 (0.26 to 2.18)	0.91 (0.31 to 2.22)	0.95 (0.28 to 2.2)	0.98 (0.39 to 1.8)	0.99 (0.33 to 2.8)	mom+dm+if										
0.42 (0.11 to 1.13)	0.46 (0.15 to 1.2)	0.87 (0.18 to 1.73)	0.92 (0.23 to 2)	0.93 (0.22 to 2.09)	0.93 (0.26 to 2.03)	0.88 (0.29 to 2.26)	0.94 (0.21 to 2.27)	0.95 (0.27 to 2.15)	0.91 (0.31 to 2.18)	0.96 (0.27 to 2.31)	0.97 (0.38 to 1.83)	0.99 (0.31 to 3)	1 (0.33 to 2.95)	mom+dm+mp+pbo									
0.38 (0.06 to 2.43)	0.42 (0.08 to 2.59)	0.73 (0.12 to 4.25)	0.79 (0.13 to 4.53)	0.8 (0.13 to 4.59)	0.82 (0.15 to 4.69)	0.82 (0.15 to 4.78)	0.81 (0.13 to 4.97)	0.83 (0.15 to 4.98)	0.85 (0.17 to 4.71)	0.85 (0.15 to 5.01)	0.87 (0.18 to 4.6)	0.92 (0.17 to 5.88)	0.94 (0.17 to 5.9)	0.95 (0.17 to 5.95)	phos								
0.35 (0.11 to 1.4)	0.39 (0.14 to 1.5)	0.68 (0.25 to 2.25)	0.72 (0.28 to 2.67)	0.72 (0.27 to 2.75)	0.73 (0.32 to 2.64)	0.75 (0.3 to 2.69)	0.74 (0.27 to 2.94)	0.74 (0.31 to 3.06)	0.77 (0.35 to 2.67)	0.75 (0.31 to 3.19)	0.78 (0.38 to 2.65)	0.8 (0.34 to 4.31)	0.81 (0.34 to 4.4)	0.81 (0.34 to 4.36)	0.95 (0.16 to 6.66)	mom+pf+lbov							
0.34 (0.12 to 1.01)	0.38 (0.15 to 1.05)	0.65 (0.24 to 1.69)	0.7 (0.3 to 1.93)	0.71 (0.28 to 2)	0.72 (0.33 to 1.91)	0.73 (0.33 to 1.78)	0.72 (0.28 to 2.2)	0.73 (0.31 to 2.21)	0.76 (0.39 to 1.68)	0.74 (0.32 to 2.41)	0.78 (0.41 to 1.77)	0.79 (0.34 to 3.03)	0.8 (0.35 to 2.99)	0.8 (0.35 to 3.14)	0.91 (0.16 to 5.1)	0.99 (0.35 to 2.15)	mom+bovc						
0.32 (0.08 to 1.48)	0.36 (0.1 to 1.66)	0.62 (0.16 to 2.45)	0.67 (0.19 to 2.83)	0.67 (0.17 to 2.94)	0.68 (0.2 to 2.97)	0.68 (0.21 to 2.98)	0.68 (0.18 to 3.21)	0.69 (0.19 to 3.31)	0.71 (0.23 to 2.91)	0.71 (0.2 to 3.4)	0.73 (0.25 to 2.95)	0.75 (0.23 to 4.56)	0.76 (0.23 to 4.62)	0.77 (0.23 to 4.49)	0.85 (0.12 to 6.65)	0.93 (0.2 to 3.43)	0.95 (0.26 to 3.65)	mom+pf+p					
0.32 (0.08 to 1.46)	0.35 (0.1 to 1.63)	0.61 (0.15 to 2.43)	0.66 (0.18 to 2.85)	0.66 (0.17 to 2.9)	0.67 (0.19 to 2.88)	0.66 (0.2 to 2.87)	0.67 (0.17 to 3.11)	0.69 (0.19 to 3.11)	0.69 (0.22 to 2.91)	0.69 (0.2 to 3.37)	0.71 (0.24 to 2.9)	0.74 (0.22 to 4.29)	0.75 (0.23 to 4.5)	0.75 (0.23 to 4.35)	0.84 (0.11 to 6.37)	0.9 (0.19 to 3.52)	0.93 (0.25 to 3.44)	0.99 (0.31 to 2.88)	mom+if				
0.32 (0.1 to 1.01)	0.35 (0.13 to 1.05)	0.61 (0.18 to 1.66)	0.64 (0.22 to 1.92)	0.65 (0.22 to 1.98)	0.66 (0.26 to 1.81)	0.67 (0.27 to 1.85)	0.67 (0.21 to 2.09)	0.68 (0.25 to 2.1)	0.69 (0.32 to 1.79)	0.69 (0.26 to 2.36)	0.71 (0.34 to 1.82)	0.73 (0.28 to 2.89)	0.74 (0.3 to 2.88)	0.74 (0.29 to 3.12)	0.83 (0.14 to 4.78)	0.94 (0.28 to 1.98)	0.96 (0.36 to 2.05)	0.97 (0.23 to 3.7)	0.99 (0.23 to 3.88)	mom+pf+hp+pbov			
0.27 (0.08 to 0.81)	0.3 (0.11 to 0.84)	0.52 (0.15 to 1.35)	0.56 (0.19 to 1.48)	0.57 (0.17 to 1.49)	0.57 (0.21 to 1.44)	0.57 (0.22 to 1.45)	0.58 (0.17 to 1.59)	0.59 (0.19 to 1.55)	0.59 (0.25 to 1.35)	0.6 (0.2 to 1.66)	0.61 (0.28 to 1.36)	0.64 (0.23 to 2.01)	0.66 (0.23 to 2.01)	0.65 (0.23 to 2.01)	0.7 (0.11 to 3.93)	0.76 (0.19 to 2.08)	0.79 (0.27 to 1.97)	0.9 (0.22 to 1.94)	0.92 (0.24 to 1.91)	0.86 (0.28 to 2.25)	mom+pf		
0.26 (0.09 to 0.61)	0.28 (0.13 to 0.6)	0.5 (0.16 to 1)	0.54 (0.2 to 1.1)	0.54 (0.19 to 1.14)	0.54 (0.25 to 1.01)	0.54 (0.27 to 1.07)	0.55 (0.19 to 1.2)	0.56 (0.22 to 1.18)	0.56 (0.32 to 0.97)	0.57 (0.23 to 1.27)	0.58 (0.36 to 0.93)	0.61 (0.26 to 1.62)	0.61 (0.27 to 1.58)	0.62 (0.27 to 1.61)	0.67 (0.12 to 3.2)	0.76 (0.22 to 1.31)	0.77 (0.32 to 1.25)	0.8 (0.19 to 2.25)	0.82 (0.19 to 2.3)	0.85 (0.34 to 1.48)	0.95 (0.44 to 2.01)	mom+pf+pbov	
0.17 (0.05 to 0.47)	0.19 (0.07 to 0.46)	0.33 (0.1 to 0.81)	0.35 (0.12 to 0.89)	0.35 (0.11 to 0.92)	0.36 (0.14 to 0.87)	0.36 (0.19 to 0.66)	0.36 (0.11 to 0.96)	0.37 (0.13 to 0.95)	0.37 (0.21 to 0.64)	0.37 (0.13 to 1.02)	0.38 (0.18 to 0.8)	0.4 (0.15 to 1.19)	0.41 (0.16 to 1.25)	0.41 (0.15 to 1.26)	0.44 (0.08 to 2.34)	0.48 (0.13 to 1.2)	0.49 (0.2 to 1.09)	0.52 (0.12 to 1.83)	0.54 (0.12 to 1.84)	0.54 (0.19 to 1.34)	0.63 (0.24 to 1.64)	0.67 (0.32 to 1.34)	pform

Random effects model for interventions

BIN - Unadjusted NMA (Odds Ratio)

mom+dm+shdf																							
0.96 (0.36 to 2.01)	mom+dm+hdf																						
0.53 (0.16 to 1.65)	0.57 (0.2 to 1.59)	mom+dm																					
0.52 (0.17 to 1.73)	0.56 (0.22 to 1.77)	0.96 (0.36 to 3.28)	mom+dm+hpfa+lf																				
0.49 (0.16 to 1.55)	0.54 (0.2 to 1.56)	0.93 (0.34 to 2.79)	0.99 (0.33 to 2.36)	mom+dm+ehlf																			
0.49 (0.16 to 1.37)	0.53 (0.21 to 1.31)	0.93 (0.47 to 1.73)	0.96 (0.31 to 2.22)	1 (0.35 to 2.4)	mom+pform																		
0.5 (0.15 to 1.54)	0.54 (0.21 to 1.51)	0.93 (0.34 to 2.81)	0.98 (0.33 to 2.27)	1 (0.37 to 2.68)	0.99 (0.42 to 2.77)	mom+dm+lf+pbov																	
0.49 (0.16 to 1.38)	0.53 (0.22 to 1.3)	0.92 (0.35 to 2.44)	0.98 (0.31 to 2.1)	0.99 (0.37 to 2.38)	0.98 (0.44 to 2.41)	1 (0.37 to 2.31)	mom+dm+ef																
0.48 (0.14 to 1.54)	0.52 (0.19 to 1.55)	0.89 (0.31 to 2.85)	0.97 (0.29 to 2.27)	0.99 (0.34 to 2.59)	0.96 (0.39 to 2.85)	0.99 (0.35 to 2.53)	0.99 (0.38 to 2.64)	mom+pdm+pbov															
0.47 (0.15 to 1.41)	0.51 (0.2 to 1.4)	0.88 (0.32 to 2.5)	0.96 (0.28 to 2.12)	0.98 (0.32 to 2.43)	0.96 (0.38 to 2.48)	0.98 (0.34 to 2.28)	0.99 (0.36 to 2.41)	0.99 (0.34 to 2.74)	mom+dm+hp+pbo														
0.47 (0.13 to 1.42)	0.5 (0.18 to 1.39)	0.87 (0.31 to 2.52)	0.95 (0.26 to 2.04)	0.97 (0.3 to 2.4)	0.93 (0.38 to 2.54)	0.98 (0.32 to 2.3)	0.98 (0.36 to 2.36)	0.99 (0.31 to 2.6)	1 (0.35 to 2.65)	mom+dm+af													
0.45 (0.16 to 1.07)	0.49 (0.23 to 1)	0.85 (0.37 to 1.84)	0.93 (0.33 to 1.5)	0.96 (0.4 to 1.68)	0.92 (0.48 to 1.78)	0.96 (0.41 to 1.59)	0.96 (0.47 to 1.61)	0.98 (0.39 to 1.85)	0.99 (0.45 to 1.86)	0.99 (0.46 to 1.95)	mom+dm+pbov												
0.44 (0.12 to 1.29)	0.48 (0.16 to 1.3)	0.82 (0.27 to 2.26)	0.92 (0.21 to 1.92)	0.94 (0.26 to 2.14)	0.89 (0.32 to 2.24)	0.95 (0.26 to 2.06)	0.95 (0.3 to 2.14)	0.96 (0.26 to 2.41)	0.97 (0.3 to 2.37)	0.98 (0.29 to 2.56)	0.99 (0.42 to 1.98)	mom+dm+lbov											
0.43 (0.1 to 1.23)	0.46 (0.14 to 1.24)	0.79 (0.25 to 2.16)	0.9 (0.18 to 1.87)	0.93 (0.21 to 2.09)	0.86 (0.3 to 2.19)	0.93 (0.23 to 1.97)	0.94 (0.26 to 2)	0.95 (0.23 to 2.38)	0.95 (0.26 to 2.22)	0.96 (0.28 to 2.32)	0.98 (0.37 to 1.86)	0.99 (0.3 to 2.77)	mom+dm+mp+pbo										
0.43 (0.11 to 1.21)	0.46 (0.15 to 1.19)	0.8 (0.26 to 2.12)	0.9 (0.21 to 1.78)	0.93 (0.24 to 1.97)	0.87 (0.31 to 2.13)	0.92 (0.25 to 1.92)	0.93 (0.28 to 2.01)	0.95 (0.25 to 2.21)	0.96 (0.28 to 2.24)	0.96 (0.31 to 2.22)	0.97 (0.41 to 1.78)	0.99 (0.31 to 2.71)	1 (0.35 to 2.92)	mom+dm+if									
0.4 (0.06 to 2.71)	0.43 (0.08 to 2.69)	0.74 (0.15 to 4.83)	0.76 (0.13 to 4.43)	0.8 (0.14 to 4.77)	0.8 (0.16 to 5.01)	0.81 (0.14 to 4.76)	0.81 (0.16 to 4.74)	0.83 (0.14 to 5.18)	0.85 (0.16 to 4.94)	0.86 (0.16 to 5.07)	0.87 (0.19 to 4.75)	0.92 (0.17 to 5.75)	0.94 (0.18 to 6.26)	0.93 (0.18 to 6.16)	phos								
0.38 (0.11 to 1.55)	0.41 (0.14 to 1.57)	0.71 (0.25 to 2.61)	0.72 (0.25 to 2.42)	0.75 (0.27 to 2.76)	0.76 (0.32 to 2.61)	0.75 (0.28 to 2.81)	0.76 (0.32 to 2.75)	0.77 (0.29 to 3.16)	0.78 (0.31 to 3.22)	0.79 (0.31 to 3.17)	0.82 (0.39 to 2.69)	0.82 (0.33 to 4.02)	0.84 (0.35 to 4.35)	0.84 (0.36 to 4.19)	0.96 (0.15 to 6.41)	mom+pf+lbov							
0.37 (0.11 to 1.18)	0.4 (0.15 to 1.14)	0.69 (0.26 to 1.89)	0.7 (0.25 to 1.84)	0.73 (0.28 to 2.12)	0.75 (0.35 to 1.76)	0.73 (0.29 to 2.05)	0.74 (0.32 to 1.98)	0.75 (0.28 to 2.32)	0.76 (0.32 to 2.36)	0.77 (0.31 to 2.38)	0.8 (0.41 to 1.86)	0.81 (0.34 to 2.89)	0.83 (0.35 to 3.32)	0.83 (0.36 to 3.02)	0.92 (0.14 to 5.43)	0.99 (0.34 to 2.22)	mom+bovc						
0.33 (0.08 to 1.54)	0.36 (0.09 to 1.71)	0.62 (0.17 to 2.78)	0.64 (0.16 to 2.52)	0.67 (0.17 to 2.82)	0.67 (0.2 to 2.85)	0.66 (0.18 to 2.83)	0.68 (0.19 to 2.94)	0.68 (0.18 to 3.29)	0.69 (0.19 to 3.28)	0.7 (0.19 to 3.32)	0.72 (0.23 to 2.98)	0.74 (0.2 to 4.1)	0.76 (0.21 to 4.49)	0.76 (0.22 to 4.24)	0.82 (0.11 to 6.56)	0.88 (0.19 to 3.49)	0.9 (0.23 to 3.65)	mom+if					
0.33 (0.08 to 1.6)	0.36 (0.09 to 1.7)	0.63 (0.16 to 2.87)	0.65 (0.15 to 2.66)	0.67 (0.18 to 2.95)	0.67 (0.2 to 2.87)	0.67 (0.18 to 2.92)	0.68 (0.19 to 2.97)	0.69 (0.18 to 3.4)	0.69 (0.19 to 3.33)	0.71 (0.2 to 3.46)	0.73 (0.23 to 3.01)	0.74 (0.21 to 4.26)	0.77 (0.22 to 4.89)	0.77 (0.22 to 4.53)	0.83 (0.11 to 6.79)	0.89 (0.19 to 3.65)	0.91 (0.23 to 3.62)	1 (0.35 to 3.01)	mom+pf+p				
0.34 (0.1 to 1.2)	0.37 (0.13 to 1.18)	0.63 (0.23 to 2)	0.65 (0.2 to 1.89)	0.69 (0.23 to 2.13)	0.69 (0.3 to 1.95)	0.69 (0.23 to 2.06)	0.7 (0.26 to 2.06)	0.71 (0.23 to 2.29)	0.72 (0.26 to 2.36)	0.72 (0.25 to 2.48)	0.74 (0.35 to 2.02)	0.76 (0.28 to 2.83)	0.77 (0.29 to 3.27)	0.77 (0.31 to 3.04)	0.86 (0.13 to 5.08)	0.95 (0.29 to 2.01)	0.97 (0.37 to 2.17)	1.04 (0.22 to 4.39)	1.03 (0.22 to 4.2)	mom+pf+hp+pbov			
0.28 (0.07 to 0.93)	0.3 (0.1 to 0.9)	0.53 (0.17 to 1.42)	0.54 (0.15 to 1.52)	0.57 (0.17 to 1.67)	0.57 (0.22 to 1.42)	0.57 (0.17 to 1.58)	0.57 (0.2 to 1.57)	0.59 (0.17 to 1.74)	0.6 (0.19 to 1.73)	0.6 (0.19 to 1.77)	0.62 (0.25 to 1.49)	0.64 (0.21 to 2)	0.66 (0.22 to 2.19)	0.66 (0.22 to 2.13)	0.7 (0.1 to 3.89)	0.73 (0.17 to 2.09)	0.75 (0.24 to 2.04)	0.92 (0.24 to 1.88)	0.92 (0.23 to 1.84)	0.82 (0.24 to 2.35)	mom+pf		
0.28 (0.09 to 0.76)	0.31 (0.13 to 0.7)	0.53 (0.23 to 1.17)	0.55 (0.17 to 1.19)	0.58 (0.21 to 1.24)	0.57 (0.31 to 1.07)	0.58 (0.21 to 1.24)	0.58 (0.25 to 1.19)	0.6 (0.2 to 1.4)	0.6 (0.23 to 1.35)	0.61 (0.24 to 1.39)	0.62 (0.36 to 1.09)	0.65 (0.26 to 1.6)	0.66 (0.27 to 1.81)	0.66 (0.29 to 1.72)	0.72 (0.12 to 3.33)	0.8 (0.23 to 1.38)	0.82 (0.32 to 1.33)	0.86 (0.2 to 2.75)	0.86 (0.19 to 2.75)	0.88 (0.32 to 1.53)	1.01 (0.42 to 2.54)	mom+pf+pbov	
0.18 (0.05 to 0.58)	0.2 (0.07 to 0.55)	0.35 (0.16 to 0.76)	0.36 (0.11 to 1)	0.38 (0.12 to 1.06)	0.37 (0.18 to 0.8)	0.37 (0.12 to 1.06)	0.38 (0.14 to 1.03)	0.39 (0.12 to 1.16)	0.39 (0.14 to 1.12)	0.4 (0.13 to 1.16)	0.41 (0.18 to 0.99)	0.42 (0.15 to 1.29)	0.43 (0.16 to 1.45)	0.43 (0.16 to 1.35)	0.46 (0.08 to 2.46)	0.49 (0.13 to 1.55)	0.5 (0.18 to 1.46)	0.56 (0.12 to 2.15)	0.55 (0.12 to 2.19)	0.54 (0.17 to 1.53)	0.66 (0.24 to 2.06)	0.66 (0.28 to 1.55)	pform

Fixed effects model for classes

BIN - Unadjusted NMA (Odds Ratio)

mom+dm+hm							
0.52 (0.16 to 1.7)	mom+dm						
0.5 (0.16 to 1.57)	0.96 (0.33 to 2.76)	mom+form					
0.49 (0.22 to 1.08)	0.94 (0.37 to 2.35)	0.97 (0.41 to 2.27)	mom+dm+bov				
0.43 (0.08 to 2.64)	0.83 (0.14 to 5.2)	0.86 (0.15 to 5.12)	0.88 (0.19 to 4.45)	phosphorus			
0.35 (0.13 to 1.07)	0.67 (0.27 to 2.06)	0.69 (0.3 to 2.03)	0.72 (0.4 to 1.54)	0.83 (0.15 to 4.45)	mom+form+bov		
0.34 (0.11 to 1.24)	0.65 (0.2 to 2.42)	0.68 (0.22 to 2.4)	0.69 (0.29 to 1.95)	0.8 (0.13 to 4.88)	0.97 (0.32 to 2.81)	mom+bov	
0.2 (0.06 to 0.73)	0.39 (0.14 to 1.3)	0.4 (0.15 to 1.35)	0.42 (0.17 to 1.19)	0.48 (0.08 to 2.83)	0.59 (0.21 to 1.66)	0.61 (0.17 to 2.14)	form

Random effects model for classes

BIN - Unadjusted NMA (Odds Ratio)

mom+dm+hm							
0.57<br>(0.16 to 1.93)	mom+dm						
0.53<br>(0.16 to 1.68)	0.93<br>(0.32 to 2.8)	mom+form					
0.49<br>(0.21 to 1.14)	0.86<br>(0.33 to 2.24)	0.92<br>(0.39 to 2.25)	mom+dm+bov				
0.43<br>(0.08 to 2.74)	0.78<br>(0.14 to 5.04)	0.83<br>(0.15 to 5.21)	0.89<br>(0.19 to 4.47)	phosphorus			
0.37<br>(0.13 to 1.14)	0.65<br>(0.23 to 2.08)	0.69<br>(0.29 to 2.12)	0.75<br>(0.4 to 1.63)	0.85<br>(0.15 to 4.55)	mom+form+bov		
0.34<br>(0.1 to 1.36)	0.61<br>(0.17 to 2.43)	0.64<br>(0.2 to 2.46)	0.7<br>(0.26 to 2.08)	0.79<br>(0.12 to 4.97)	0.92<br>(0.29 to 2.81)	mom+bov	
0.22<br>(0.07 to 0.83)	0.38<br>(0.13 to 1.39)	0.41<br>(0.14 to 1.46)	0.44<br>(0.18 to 1.3)	0.49<br>(0.08 to 2.94)	0.59<br>(0.19 to 1.84)	0.63<br>(0.17 to 2.45)	form

ROP

Fixed effects model for interventions

BIN - Unadjusted NMA (Odds Ratio)

phos																
0.87 (0.15 to 4.76)	mom+pform															
0.92 (0.13 to 6.12)	1.07 (0.33 to 3.54)	mom+pf														
0.58 (0.13 to 2.42)	0.67 (0.22 to 2.09)	0.63 (0.16 to 2.58)	mom+dm+af													
0.6 (0.1 to 3.64)	0.69 (0.35 to 1.4)	0.65 (0.17 to 2.46)	1.05 (0.32 to 3.28)	mom+dm												
0.56 (0.13 to 2.21)	0.64 (0.21 to 1.89)	0.6 (0.16 to 2.31)	0.99 (0.49 to 1.59)	0.92 (0.3 to 2.9)	mom+dm+hpfa+lf											
0.56 (0.12 to 2.26)	0.64 (0.18 to 2.28)	0.59 (0.14 to 2.7)	0.96 (0.4 to 2.16)	0.92 (0.25 to 3.47)	1 (0.44 to 2.24)	pform										
0.55 (0.12 to 2.27)	0.63 (0.2 to 1.98)	0.59 (0.15 to 2.35)	0.98 (0.44 to 1.64)	0.9 (0.28 to 3)	1 (0.53 to 1.77)	0.99 (0.42 to 2.31)	mom+pdm+pbov									
0.54 (0.12 to 2.1)	0.63 (0.21 to 1.85)	0.58 (0.15 to 2.25)	0.98 (0.46 to 1.55)	0.9 (0.29 to 2.81)	0.99 (0.55 to 1.65)	0.99 (0.46 to 2.08)	1 (0.54 to 1.89)	mom+dm+ef								
0.55 (0.13 to 2.1)	0.63 (0.22 to 1.76)	0.58 (0.16 to 2.19)	0.98 (0.53 to 1.32)	0.9 (0.3 to 2.7)	0.99 (0.65 to 1.39)	0.99 (0.46 to 2.03)	1 (0.61 to 1.59)	1 (0.67 to 1.49)	mom+dm+pbov							
0.55 (0.12 to 2.18)	0.62 (0.2 to 1.89)	0.58 (0.15 to 2.33)	0.98 (0.43 to 1.56)	0.9 (0.28 to 2.9)	0.99 (0.52 to 1.71)	0.99 (0.41 to 2.21)	1 (0.5 to 1.93)	1 (0.54 to 1.79)	1 (0.63 to 1.58)	mom+dm+lf+pbov						
0.53 (0.12 to 2.14)	0.61 (0.19 to 1.83)	0.57 (0.14 to 2.23)	0.96 (0.43 to 1.47)	0.87 (0.27 to 2.78)	0.98 (0.5 to 1.61)	0.96 (0.4 to 2.17)	0.99 (0.48 to 1.75)	0.99 (0.51 to 1.72)	0.99 (0.61 to 1.47)	1 (0.5 to 1.76)	mom+dm+if					
0.53 (0.11 to 2.12)	0.6 (0.19 to 1.82)	0.56 (0.14 to 2.22)	0.96 (0.37 to 1.46)	0.86 (0.26 to 2.85)	0.98 (0.46 to 1.59)	0.95 (0.37 to 2.14)	0.99 (0.43 to 1.74)	0.99 (0.48 to 1.68)	0.99 (0.55 to 1.44)	0.99 (0.47 to 1.74)	1 (0.5 to 1.83)	mom+dm+hp+pbov				
0.42 (0.1 to 1.62)	0.49 (0.16 to 1.52)	0.45 (0.12 to 1.85)	0.73 (0.35 to 1.4)	0.7 (0.22 to 2.46)	0.76 (0.4 to 1.51)	0.77 (0.34 to 1.68)	0.77 (0.39 to 1.66)	0.78 (0.46 to 1.36)	0.78 (0.45 to 1.38)	0.78 (0.4 to 1.63)	0.8 (0.42 to 1.73)	0.8 (0.42 to 1.84)	mom+pf+pbov			
0.41 (0.09 to 1.69)	0.48 (0.14 to 1.59)	0.44 (0.11 to 1.9)	0.72 (0.31 to 1.53)	0.69 (0.21 to 2.52)	0.75 (0.35 to 1.6)	0.76 (0.31 to 1.79)	0.76 (0.35 to 1.77)	0.76 (0.4 to 1.52)	0.76 (0.4 to 1.52)	0.76 (0.36 to 1.76)	0.78 (0.37 to 1.83)	0.79 (0.38 to 1.95)	0.99 (0.6 to 1.57)	mom+pf+hp+pbov		
0.39 (0.09 to 1.55)	0.45 (0.14 to 1.43)	0.41 (0.1 to 1.73)	0.68 (0.27 to 1.36)	0.64 (0.19 to 2.36)	0.71 (0.33 to 1.41)	0.71 (0.29 to 1.63)	0.72 (0.32 to 1.45)	0.72 (0.36 to 1.36)	0.72 (0.39 to 1.32)	0.72 (0.33 to 1.48)	0.74 (0.34 to 1.54)	0.75 (0.36 to 1.61)	0.97 (0.49 to 1.44)	0.98 (0.46 to 1.58)	mom+bovc	
0.35 (0.08 to 1.54)	0.4 (0.12 to 1.3)	0.37 (0.09 to 1.62)	0.6 (0.26 to 1.24)	0.58 (0.17 to 1.97)	0.63 (0.3 to 1.31)	0.63 (0.29 to 1.38)	0.64 (0.3 to 1.39)	0.64 (0.36 to 1.18)	0.64 (0.35 to 1.22)	0.65 (0.31 to 1.4)	0.66 (0.32 to 1.45)	0.66 (0.32 to 1.54)	0.82 (0.44 to 1.52)	0.84 (0.4 to 1.77)	0.88 (0.43 to 1.95)	mom+dm+hdf

Random effects model for interventions

BIN - Unadjusted NMA (Odds Ratio)

phos																
0.87 (0.08 to 6.53)	mom+pf															
0.81 (0.11 to 5.26)	0.9 (0.25 to 3.45)	mom+pform														
0.58 (0.12 to 2.61)	0.64 (0.16 to 3.17)	0.69 (0.22 to 2.67)	mom+dm+af													
0.55 (0.11 to 2.35)	0.61 (0.15 to 2.92)	0.67 (0.21 to 2.34)	0.99 (0.46 to 1.65)	mom+dm+hpfa+lf												
0.58 (0.08 to 3.78)	0.64 (0.16 to 2.98)	0.7 (0.32 to 1.71)	1.01 (0.27 to 3.47)	1.05 (0.3 to 3.64)	mom+dm											
0.54 (0.12 to 2.21)	0.6 (0.15 to 2.78)	0.65 (0.22 to 2.15)	0.98 (0.5 to 1.36)	0.99 (0.64 to 1.46)	0.92 (0.28 to 3.06)	mom+dm+pbov										
0.54 (0.11 to 2.43)	0.6 (0.14 to 2.98)	0.65 (0.2 to 2.41)	0.98 (0.4 to 1.74)	0.99 (0.49 to 1.89)	0.93 (0.26 to 3.36)	1 (0.59 to 1.71)	mom+pdm+pbov									
0.53 (0.11 to 2.32)	0.59 (0.14 to 2.92)	0.64 (0.2 to 2.32)	0.97 (0.4 to 1.61)	0.99 (0.49 to 1.81)	0.91 (0.27 to 3.29)	1 (0.6 to 1.59)	1 (0.48 to 2.04)	mom+dm+ef								
0.54 (0.1 to 2.44)	0.59 (0.13 to 3.16)	0.65 (0.18 to 2.72)	0.95 (0.34 to 2.24)	0.98 (0.39 to 2.34)	0.93 (0.23 to 4)	1 (0.44 to 2.2)	1 (0.39 to 2.48)	1.02 (0.42 to 2.26)	pform							
0.54 (0.1 to 2.37)	0.6 (0.14 to 2.93)	0.65 (0.2 to 2.29)	0.98 (0.4 to 1.63)	0.99 (0.5 to 1.8)	0.93 (0.25 to 3.26)	1 (0.59 to 1.59)	1 (0.47 to 1.99)	1 (0.5 to 2.04)	1 (0.4 to 2.55)	mom+dm+lf+pbov						
0.52 (0.11 to 2.3)	0.58 (0.14 to 2.87)	0.63 (0.19 to 2.31)	0.96 (0.4 to 1.52)	0.98 (0.47 to 1.73)	0.9 (0.25 to 3.28)	0.99 (0.59 to 1.51)	0.99 (0.45 to 1.89)	0.99 (0.49 to 1.88)	0.96 (0.39 to 2.48)	0.99 (0.48 to 1.87)	mom+dm+if					
0.51 (0.1 to 2.33)	0.58 (0.12 to 2.78)	0.62 (0.17 to 2.2)	0.96 (0.35 to 1.55)	0.98 (0.42 to 1.67)	0.89 (0.23 to 3.13)	0.99 (0.52 to 1.56)	0.99 (0.41 to 1.84)	0.99 (0.43 to 1.89)	0.96 (0.34 to 2.49)	0.99 (0.44 to 1.82)	1 (0.47 to 1.91)	mom+dm+hp+pbov				
0.43 (0.08 to 1.81)	0.45 (0.1 to 2.27)	0.49 (0.16 to 1.85)	0.72 (0.29 to 1.53)	0.75 (0.36 to 1.63)	0.71 (0.2 to 2.64)	0.76 (0.41 to 1.51)	0.75 (0.35 to 1.74)	0.77 (0.42 to 1.46)	0.76 (0.34 to 1.73)	0.76 (0.35 to 1.77)	0.78 (0.37 to 1.79)	0.78 (0.36 to 2)	mom+pf+pbov			
0.41 (0.08 to 1.89)	0.45 (0.1 to 2.42)	0.49 (0.14 to 1.96)	0.71 (0.26 to 1.61)	0.74 (0.31 to 1.69)	0.7 (0.19 to 2.72)	0.75 (0.35 to 1.61)	0.74 (0.31 to 1.95)	0.75 (0.35 to 1.64)	0.75 (0.3 to 1.89)	0.75 (0.31 to 1.87)	0.77 (0.33 to 1.94)	0.77 (0.32 to 2.15)	0.99 (0.58 to 1.64)	mom+pf+hp+pbov		
0.39 (0.07 to 1.75)	0.42 (0.1 to 2.2)	0.46 (0.13 to 1.69)	0.67 (0.24 to 1.47)	0.7 (0.29 to 1.5)	0.66 (0.18 to 2.39)	0.71 (0.34 to 1.41)	0.71 (0.28 to 1.6)	0.72 (0.32 to 1.5)	0.71 (0.27 to 1.73)	0.71 (0.3 to 1.59)	0.74 (0.31 to 1.62)	0.74 (0.31 to 1.77)	0.97 (0.46 to 1.55)	0.98 (0.44 to 1.63)	mom+bovc	
0.35 (0.06 to 1.58)	0.39 (0.08 to 2.04)	0.42 (0.12 to 1.72)	0.61 (0.23 to 1.4)	0.63 (0.26 to 1.45)	0.59 (0.16 to 2.37)	0.65 (0.3 to 1.38)	0.64 (0.27 to 1.59)	0.65 (0.32 to 1.33)	0.64 (0.26 to 1.52)	0.64 (0.27 to 1.58)	0.66 (0.28 to 1.59)	0.67 (0.27 to 1.74)	0.84 (0.41 to 1.76)	0.84 (0.37 to 2.07)	0.9 (0.4 to 2.23)	mom+dm+hdf

Fixed effects model for classes

BIN - Unadjusted NMA (Odds Ratio)

phosphorus							
0.93 (0.13 to 5.93)	mom+bov						
0.87 (0.14 to 4.79)	0.94 (0.25 to 3.34)	mom+form					
0.61 (0.1 to 3.65)	0.66 (0.16 to 2.69)	0.7 (0.3 to 1.78)	mom+dm				
0.58 (0.12 to 2.5)	0.6 (0.14 to 2.93)	0.65 (0.17 to 2.52)	0.92 (0.24 to 3.62)	form			
0.56 (0.13 to 2.11)	0.6 (0.17 to 2.22)	0.64 (0.22 to 1.9)	0.9 (0.29 to 2.76)	0.98 (0.43 to 2.2)	mom+dm+bov		
0.42 (0.1 to 1.72)	0.45 (0.11 to 1.9)	0.48 (0.15 to 1.61)	0.68 (0.2 to 2.47)	0.75 (0.3 to 1.84)	0.76 (0.42 to 1.39)	mom+form+bov	
0.36 (0.08 to 1.74)	0.39 (0.09 to 1.75)	0.42 (0.12 to 1.54)	0.59 (0.16 to 2.24)	0.64 (0.25 to 1.75)	0.65 (0.32 to 1.4)	0.85 (0.4 to 1.96)	mom+dm+hm

Random effects model for classes

BIN - Unadjusted NMA (Odds Ratio)

phosphorus							
0.87 (0.09 to 6.61)	mom+bov						
0.82 (0.11 to 5.31)	0.92 (0.24 to 3.82)	mom+form					
0.59 (0.08 to 3.94)	0.65 (0.15 to 3.22)	0.71 (0.26 to 2.15)	mom+dm				
0.56 (0.1 to 2.78)	0.62 (0.13 to 3.53)	0.67 (0.17 to 2.98)	0.93 (0.22 to 4.28)	form			
0.55 (0.13 to 2.31)	0.62 (0.16 to 2.81)	0.66 (0.22 to 2.29)	0.93 (0.28 to 3.13)	0.99 (0.41 to 2.48)	mom+dm+bov		
0.42 (0.08 to 1.93)	0.46 (0.11 to 2.41)	0.5 (0.15 to 1.95)	0.7 (0.19 to 2.67)	0.74 (0.28 to 1.95)	0.74 (0.38 to 1.49)	mom+form+bov	
0.36 (0.06 to 1.88)	0.41 (0.08 to 2.33)	0.44 (0.12 to 1.93)	0.62 (0.15 to 2.63)	0.66 (0.23 to 1.88)	0.65 (0.29 to 1.58)	0.87 (0.37 to 2.29)	mom+dm+hm

Sepsis

Fixed effects model for interventions

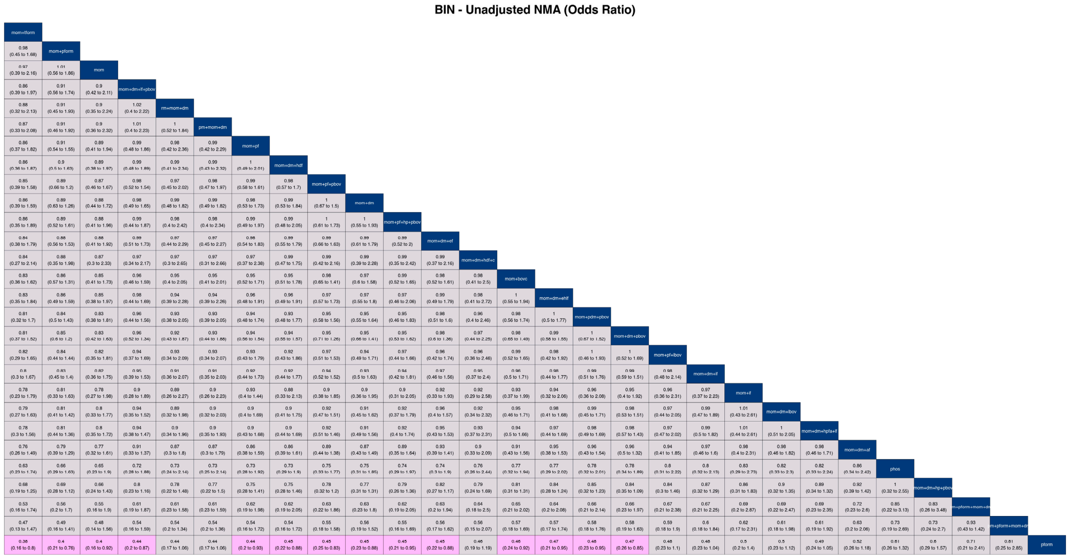

Random effects model for interventions

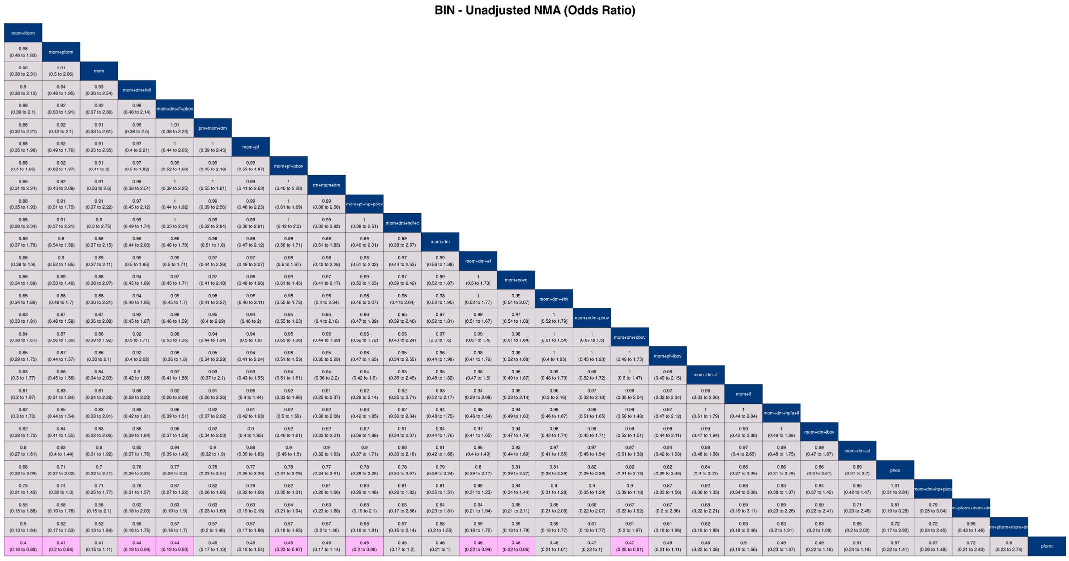

Fixed effects model for classes

BIN - Unadjusted NMA (Odds Ratio)

mom+form									
0.99 (0.41 to 2.33)	mom								
0.89 (0.42 to 1.82)	0.9 (0.36 to 2.32)	mom+dm							
0.87 (0.37 to 1.98)	0.88 (0.31 to 2.4)	0.98 (0.4 to 2.29)	mom+dm+hm						
0.86 (0.44 to 1.56)	0.86 (0.37 to 2.03)	0.96 (0.49 to 1.87)	0.98 (0.46 to 2.09)	mom+form+bov					
0.84 (0.35 to 1.77)	0.84 (0.3 to 2.23)	0.94 (0.38 to 2.14)	0.96 (0.38 to 2.36)	0.97 (0.47 to 1.98)	mom+bov				
0.83 (0.46 to 1.43)	0.84 (0.38 to 1.89)	0.93 (0.52 to 1.67)	0.95 (0.49 to 1.86)	0.97 (0.64 to 1.48)	1 (0.53 to 1.95)	mom+dm+bov			
0.65 (0.24 to 1.89)	0.66 (0.21 to 2.28)	0.74 (0.26 to 2.2)	0.75 (0.26 to 2.32)	0.77 (0.3 to 2.11)	0.79 (0.27 to 2.45)	0.79 (0.32 to 2.06)	phosphorus		
0.53 (0.17 to 1.62)	0.53 (0.15 to 1.92)	0.59 (0.22 to 1.54)	0.6 (0.17 to 2.07)	0.61 (0.2 to 1.82)	0.62 (0.18 to 2.18)	0.63 (0.21 to 1.8)	0.79 (0.19 to 3.02)	mom+dm+form	
0.41 (0.18 to 0.98)	0.41 (0.15 to 1.23)	0.46 (0.18 to 1.18)	0.47 (0.19 to 1.25)	0.48 (0.22 to 1.1)	0.49 (0.19 to 1.37)	0.49 (0.24 to 1.04)	0.62 (0.23 to 1.72)	0.77 (0.23 to 2.86)	form

Random effects model for classes

BIN - Unadjusted NMA (Odds Ratio)

mom+form									
0.99 (0.39 to 2.39)	mom								
0.92 (0.37 to 2.18)	0.93 (0.31 to 2.83)	mom+dm+hm							
0.9 (0.41 to 1.96)	0.91 (0.33 to 2.57)	0.99 (0.4 to 2.45)	mom+dm						
0.88 (0.44 to 1.63)	0.89 (0.35 to 2.19)	0.97 (0.43 to 2.13)	0.98 (0.47 to 2.02)	mom+form+bov					
0.85 (0.47 to 1.54)	0.87 (0.36 to 2.08)	0.94 (0.46 to 1.94)	0.95 (0.51 to 1.75)	0.97 (0.61 to 1.59)	mom+dm+bov				
0.87 (0.35 to 1.98)	0.87 (0.28 to 2.56)	0.95 (0.33 to 2.47)	0.97 (0.36 to 2.29)	0.98 (0.42 to 2.13)	1.01 (0.45 to 2.02)	mom+bov			
0.71 (0.24 to 2.2)	0.71 (0.2 to 2.67)	0.78 (0.24 to 2.64)	0.8 (0.24 to 2.58)	0.8 (0.29 to 2.4)	0.83 (0.3 to 2.33)	0.83 (0.25 to 2.89)	phosphorus		
0.54 (0.17 to 1.75)	0.56 (0.14 to 2.09)	0.6 (0.17 to 2.09)	0.6 (0.23 to 1.58)	0.62 (0.2 to 1.9)	0.64 (0.23 to 1.76)	0.63 (0.18 to 2.35)	0.76 (0.18 to 3.15)	mom+dm+form	
0.42 (0.17 to 1.03)	0.42 (0.14 to 1.35)	0.46 (0.18 to 1.26)	0.47 (0.18 to 1.24)	0.47 (0.21 to 1.12)	0.49 (0.23 to 1.06)	0.49 (0.18 to 1.47)	0.59 (0.2 to 1.74)	0.77 (0.22 to 2.71)	form

PVL

The PVL network consists of two distinct networks without any shared classes.

Fixed effects model for interventions (Network 1)

BIN - Unadjusted NMA (Odds Ratio)

mom			
0.92 (0.15 to 6.26)	pform		
0.81 (0.17 to 4.22)	0.87 (0.17 to 4)	mom+pf+pbov	
0.54 (0.25 to 1.12)	0.58 (0.09 to 3.09)	0.66 (0.15 to 2.64)	mom+pform

Random effects model for interventions (Network 1)

BIN - Unadjusted NMA (Odds Ratio)

mom			
0.87 (0.1 to 8.37)	pform		
0.81 (0.11 to 6.2)	0.91 (0.13 to 6.26)	mom+pf+pbov	
0.56 (0.18 to 1.99)	0.66 (0.09 to 4.28)	0.72 (0.14 to 3.36)	mom+pform

Fixed effects model for classes (Network 1)

BIN - Unadjusted NMA (Odds Ratio)

mom			
0.91<br>(0.14 to 6.84)	form		
0.83<br>(0.14 to 5.45)	0.91<br>(0.14 to 5.8)	mom+form+bov	
0.61<br>(0.21 to 2.25)	0.68<br>(0.14 to 3.29)	0.75<br>(0.18 to 3.16)	mom+form

Random effects model for classes (Network 1)

BIN - Unadjusted NMA (Odds Ratio)

mom			
0.87<br>(0.11 to 7.89)	form		
0.83<br>(0.11 to 6.95)	0.95<br>(0.13 to 7.09)	mom+form+bov	
0.65<br>(0.18 to 2.93)	0.76<br>(0.14 to 3.99)	0.8<br>(0.17 to 3.6)	mom+form

Fixed effects model for interventions (Network 2)

BIN - Unadjusted NMA (Odds Ratio)

mom+dm+lf+pbov		
0.95<br>(0.21 to 2.49)	mom+dm+pbov	
0.83<br>(0.16 to 2.71)	0.92<br>(0.45 to 1.85)	mom+dm+hdf

Random effects model for interventions (Network 2)

BIN - Unadjusted NMA (Odds Ratio)

mom+dm+lf+pbov		
0.95<br>(0.19 to 2.78)	mom+dm+pbov	
0.84<br>(0.13 to 4.18)	0.93<br>(0.3 to 3.08)	mom+dm+hdf

Fixed effects model for classes (Network 2)

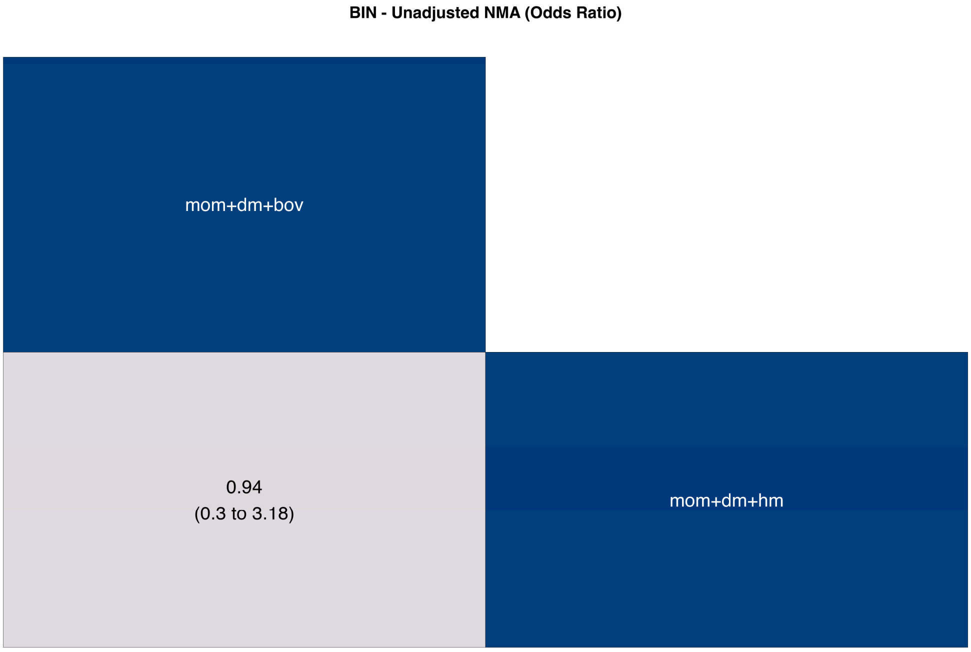

Random effects model for classes (Network 2)

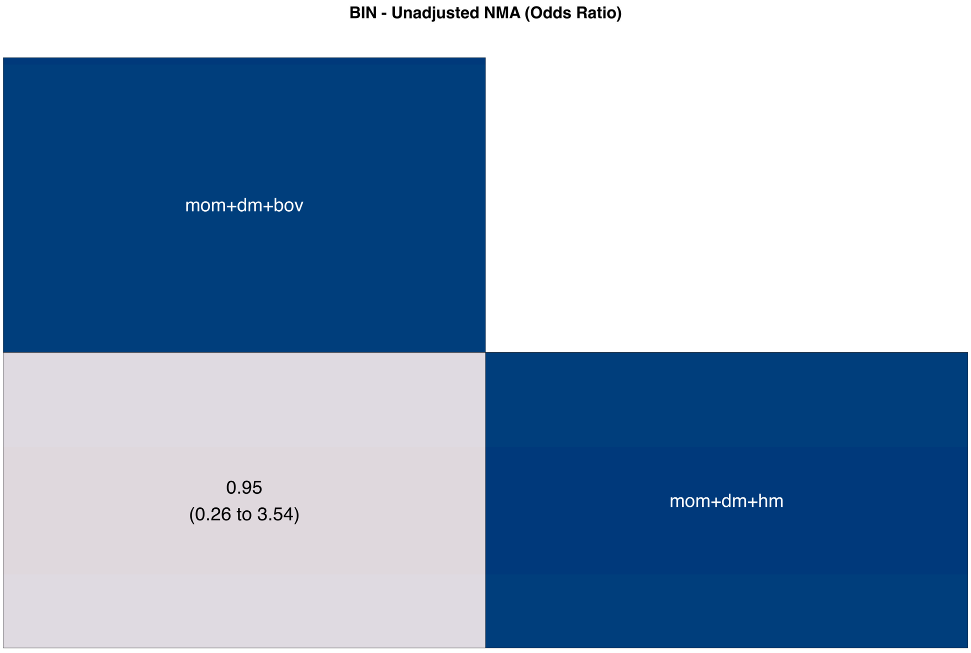

BPD

Fixed effects model for interventions

BIN - Unadjusted NMA (Odds Ratio)

phos																		
0.79 (0.25 to 2.26)	mom+dm+hdf+c																	
0.74 (0.25 to 1.94)	0.98 (0.56 to 1.37)	mom+dm+hdf																
0.64 (0.22 to 1.6)	0.82 (0.39 to 1.57)	0.86 (0.49 to 1.54)	mom+dm+hpfa+lf															
0.63 (0.21 to 1.62)	0.81 (0.36 to 1.56)	0.84 (0.46 to 1.52)	1 (0.6 to 1.5)	mom+dm+ehlf														
0.62 (0.21 to 1.58)	0.8 (0.36 to 1.54)	0.84 (0.46 to 1.51)	1 (0.6 to 1.47)	1 (0.6 to 1.6)	mom+dm+hp+pbov													
0.62 (0.21 to 1.53)	0.8 (0.39 to 1.44)	0.84 (0.5 to 1.39)	0.99 (0.61 to 1.45)	1 (0.61 to 1.59)	1 (0.63 to 1.61)	mom+dm+ef												
0.62 (0.22 to 1.5)	0.79 (0.38 to 1.43)	0.83 (0.5 to 1.37)	0.99 (0.68 to 1.25)	1 (0.68 to 1.37)	1 (0.7 to 1.37)	1 (0.71 to 1.35)	mom+dm+pbov											
0.61 (0.2 to 1.56)	0.79 (0.34 to 1.51)	0.82 (0.44 to 1.46)	0.99 (0.55 to 1.43)	1 (0.56 to 1.55)	1 (0.58 to 1.56)	1 (0.58 to 1.54)	1 (0.67 to 1.39)	mom+dm+if										
0.61 (0.2 to 1.55)	0.79 (0.34 to 1.49)	0.83 (0.44 to 1.46)	0.99 (0.56 to 1.43)	1 (0.57 to 1.52)	1 (0.59 to 1.53)	1 (0.59 to 1.51)	1 (0.69 to 1.36)	1 (0.62 to 1.61)	mom+dm+af									
0.61 (0.2 to 1.53)	0.79 (0.34 to 1.5)	0.83 (0.44 to 1.47)	0.99 (0.56 to 1.44)	0.99 (0.61 to 1.46)	1 (0.58 to 1.54)	1 (0.59 to 1.53)	1 (0.69 to 1.38)	1 (0.61 to 1.66)	1 (0.62 to 1.64)	mom+dm+lbov								
0.61 (0.23 to 1.45)	0.77 (0.29 to 1.96)	0.82 (0.35 to 1.95)	0.96 (0.42 to 2.11)	0.98 (0.42 to 2.21)	0.99 (0.44 to 2.19)	0.99 (0.45 to 2.16)	0.99 (0.46 to 2.09)	1 (0.45 to 2.29)	1.01 (0.45 to 2.31)	1.01 (0.44 to 2.29)	pform							
0.59 (0.19 to 1.47)	0.76 (0.3 to 1.43)	0.79 (0.39 to 1.4)	0.97 (0.47 to 1.31)	0.98 (0.48 to 1.38)	0.98 (0.5 to 1.4)	0.98 (0.5 to 1.39)	0.98 (0.58 to 1.26)	0.99 (0.52 to 1.47)	0.99 (0.53 to 1.47)	0.99 (0.53 to 1.45)	0.96 (0.4 to 2.13)	mom+pdm+pbov						
0.56 (0.21 to 1.32)	0.71 (0.35 to 1.36)	0.75 (0.43 to 1.3)	0.87 (0.52 to 1.47)	0.88 (0.52 to 1.6)	0.89 (0.53 to 1.58)	0.89 (0.57 to 1.44)	0.9 (0.59 to 1.45)	0.9 (0.54 to 1.69)	0.9 (0.55 to 1.67)	0.9 (0.55 to 1.65)	0.91 (0.44 to 1.91)	0.93 (0.58 to 1.93)	mom+pf+pbov					
0.56 (0.19 to 1.41)	0.71 (0.31 to 1.47)	0.74 (0.39 to 1.46)	0.86 (0.47 to 1.61)	0.87 (0.47 to 1.73)	0.88 (0.48 to 1.72)	0.88 (0.51 to 1.62)	0.89 (0.53 to 1.63)	0.89 (0.5 to 1.86)	0.89 (0.5 to 1.85)	0.89 (0.5 to 1.83)	0.9 (0.42 to 2.09)	0.92 (0.53 to 2.17)	1 (0.65 to 1.51)	mom+pf+hp+pbov				
0.52 (0.18 to 1.27)	0.67 (0.28 to 1.3)	0.7 (0.36 to 1.27)	0.82 (0.43 to 1.36)	0.83 (0.43 to 1.43)	0.83 (0.45 to 1.43)	0.83 (0.47 to 1.38)	0.83 (0.51 to 1.34)	0.84 (0.46 to 1.49)	0.84 (0.47 to 1.48)	0.85 (0.46 to 1.48)	0.84 (0.38 to 1.86)	0.88 (0.51 to 1.62)	0.97 (0.54 to 1.28)	0.98 (0.51 to 1.36)	mom+bovc			
0.44 (0.12 to 1.7)	0.56 (0.18 to 1.82)	0.59 (0.21 to 1.84)	0.69 (0.25 to 2.08)	0.71 (0.25 to 2.2)	0.71 (0.26 to 2.18)	0.71 (0.26 to 2.11)	0.72 (0.27 to 2.14)	0.73 (0.26 to 2.3)	0.73 (0.26 to 2.27)	0.73 (0.26 to 2.26)	0.72 (0.23 to 2.53)	0.77 (0.27 to 2.4)	0.79 (0.29 to 2.25)	0.8 (0.28 to 2.43)	0.86 (0.31 to 2.6)	mom		
0.37 (0.12 to 1.14)	0.48 (0.18 to 1.23)	0.51 (0.21 to 1.22)	0.59 (0.26 to 1.35)	0.6 (0.26 to 1.42)	0.6 (0.26 to 1.41)	0.6 (0.27 to 1.34)	0.61 (0.28 to 1.34)	0.61 (0.27 to 1.47)	0.61 (0.27 to 1.46)	0.61 (0.27 to 1.45)	0.61 (0.23 to 1.63)	0.64 (0.28 to 1.62)	0.68 (0.32 to 1.37)	0.68 (0.3 to 1.51)	0.72 (0.33 to 1.65)	0.85 (0.31 to 2.23)	mom+dm	
0.38 (0.13 to 1.09)	0.49 (0.2 to 1.13)	0.52 (0.24 to 1.11)	0.61 (0.28 to 1.23)	0.62 (0.29 to 1.3)	0.62 (0.29 to 1.3)	0.62 (0.31 to 1.23)	0.63 (0.32 to 1.22)	0.63 (0.3 to 1.35)	0.63 (0.3 to 1.34)	0.63 (0.3 to 1.34)	0.63 (0.25 to 1.57)	0.66 (0.32 to 1.5)	0.69 (0.37 to 1.23)	0.7 (0.33 to 1.38)	0.74 (0.37 to 1.52)	0.87 (0.34 to 2.03)	1.02 (0.65 to 1.61)	mom+pform

Random effects model for interventions

BIN - Unadjusted NMA (Odds Ratio)

phos																		
0.75 (0.24 to 2.4)	mom+dm+hdf+c																	
0.72 (0.24 to 2.06)	0.98 (0.56 to 1.42)	mom+dm+hdf																
0.61 (0.23 to 1.66)	0.82 (0.38 to 1.7)	0.86 (0.46 to 1.62)	mom+dm+hpfa+lf															
0.59 (0.22 to 1.63)	0.8 (0.36 to 1.67)	0.84 (0.44 to 1.61)	0.99 (0.57 to 1.54)	mom+dm+hp+pbov														
0.6 (0.22 to 1.64)	0.8 (0.35 to 1.66)	0.84 (0.43 to 1.61)	0.99 (0.57 to 1.53)	1 (0.61 to 1.62)	mom+dm+ehlf													
0.6 (0.22 to 1.63)	0.8 (0.37 to 1.61)	0.84 (0.47 to 1.52)	0.99 (0.6 to 1.51)	1 (0.61 to 1.65)	1 (0.61 to 1.72)	mom+dm+ef												
0.59 (0.22 to 1.53)	0.79 (0.36 to 1.56)	0.83 (0.47 to 1.48)	0.99 (0.66 to 1.29)	1 (0.68 to 1.37)	1 (0.68 to 1.4)	1 (0.67 to 1.36)	mom+dm+pbov											
0.59 (0.23 to 1.61)	0.79 (0.28 to 2.11)	0.84 (0.33 to 2.07)	0.97 (0.41 to 2.21)	0.99 (0.42 to 2.37)	1 (0.42 to 2.34)	0.99 (0.44 to 2.27)	1.01 (0.45 to 2.21)	pform										
0.59 (0.21 to 1.6)	0.79 (0.32 to 1.64)	0.83 (0.41 to 1.6)	0.99 (0.54 to 1.47)	1 (0.56 to 1.58)	1 (0.57 to 1.62)	1 (0.57 to 1.56)	1 (0.67 to 1.4)	0.99 (0.41 to 2.36)	mom+dm+if									
0.59 (0.21 to 1.61)	0.79 (0.34 to 1.62)	0.83 (0.42 to 1.58)	0.99 (0.54 to 1.48)	0.99 (0.58 to 1.56)	1 (0.57 to 1.57)	1 (0.56 to 1.56)	1 (0.68 to 1.42)	0.99 (0.42 to 2.33)	1 (0.62 to 1.66)	mom+dm+af								
0.58 (0.21 to 1.61)	0.79 (0.33 to 1.62)	0.82 (0.41 to 1.57)	0.98 (0.53 to 1.45)	0.99 (0.57 to 1.55)	0.99 (0.6 to 1.49)	0.99 (0.55 to 1.52)	1 (0.68 to 1.37)	0.98 (0.4 to 2.32)	1 (0.59 to 1.64)	1 (0.58 to 1.64)	mom+dm+lbov							
0.56 (0.19 to 1.55)	0.76 (0.29 to 1.57)	0.79 (0.38 to 1.52)	0.97 (0.47 to 1.35)	0.98 (0.49 to 1.41)	0.98 (0.48 to 1.43)	0.98 (0.49 to 1.42)	0.98 (0.57 to 1.27)	0.94 (0.37 to 2.23)	0.99 (0.5 to 1.5)	0.99 (0.52 to 1.48)	0.99 (0.51 to 1.48)	mom+pdm+pbov						
0.54 (0.21 to 1.45)	0.73 (0.32 to 1.51)	0.76 (0.39 to 1.43)	0.88 (0.5 to 1.62)	0.9 (0.51 to 1.72)	0.9 (0.5 to 1.76)	0.9 (0.54 to 1.58)	0.91 (0.56 to 1.56)	0.91 (0.42 to 2.03)	0.91 (0.52 to 1.83)	0.91 (0.52 to 1.8)	0.91 (0.52 to 1.87)	0.95 (0.54 to 2.04)	mom+pf+pbov					
0.54 (0.2 to 1.51)	0.72 (0.29 to 1.63)	0.75 (0.36 to 1.57)	0.87 (0.44 to 1.76)	0.89 (0.45 to 1.84)	0.89 (0.45 to 1.86)	0.89 (0.48 to 1.77)	0.9 (0.5 to 1.75)	0.9 (0.38 to 2.2)	0.9 (0.47 to 1.96)	0.9 (0.47 to 1.97)	0.91 (0.47 to 1.98)	0.94 (0.5 to 2.24)	1 (0.63 to 1.54)	mom+pf+hp+pbov				
0.5 (0.18 to 1.38)	0.68 (0.27 to 1.45)	0.71 (0.34 to 1.39)	0.82 (0.42 to 1.49)	0.84 (0.43 to 1.54)	0.84 (0.43 to 1.55)	0.84 (0.44 to 1.48)	0.85 (0.49 to 1.43)	0.84 (0.35 to 2.01)	0.86 (0.45 to 1.61)	0.85 (0.45 to 1.6)	0.86 (0.45 to 1.61)	0.89 (0.49 to 1.72)	0.97 (0.52 to 1.32)	0.98 (0.49 to 1.4)	mom+bovc			
0.44 (0.11 to 1.76)	0.59 (0.17 to 2.04)	0.62 (0.2 to 2.03)	0.72 (0.24 to 2.21)	0.75 (0.24 to 2.27)	0.74 (0.23 to 2.29)	0.74 (0.24 to 2.23)	0.75 (0.25 to 2.21)	0.74 (0.21 to 2.61)	0.76 (0.24 to 2.36)	0.76 (0.24 to 2.31)	0.76 (0.25 to 2.34)	0.8 (0.26 to 2.49)	0.82 (0.29 to 2.3)	0.83 (0.28 to 2.47)	0.89 (0.31 to 2.68)	mom		
0.38 (0.12 to 1.31)	0.5 (0.17 to 1.53)	0.53 (0.2 to 1.51)	0.61 (0.26 to 1.64)	0.63 (0.26 to 1.69)	0.63 (0.27 to 1.72)	0.63 (0.27 to 1.67)	0.63 (0.28 to 1.66)	0.63 (0.22 to 1.94)	0.64 (0.27 to 1.77)	0.64 (0.27 to 1.77)	0.65 (0.27 to 1.78)	0.67 (0.28 to 1.94)	0.7 (0.3 to 1.66)	0.7 (0.28 to 1.8)	0.75 (0.31 to 2)	0.86 (0.28 to 2.62)	mom+dm	
0.37 (0.13 to 1.17)	0.5 (0.2 to 1.33)	0.53 (0.23 to 1.33)	0.61 (0.3 to 1.38)	0.63 (0.3 to 1.49)	0.63 (0.3 to 1.52)	0.63 (0.31 to 1.4)	0.63 (0.33 to 1.42)	0.63 (0.25 to 1.68)	0.64 (0.31 to 1.52)	0.64 (0.31 to 1.6)	0.64 (0.31 to 1.57)	0.66 (0.32 to 1.73)	0.7 (0.37 to 1.34)	0.7 (0.34 to 1.54)	0.75 (0.38 to 1.77)	0.86 (0.34 to 2.13)	0.99 (0.54 to 1.84)	mom+pform

Fixed effects model for classes

BIN - Unadjusted NMA (Odds Ratio)

phosphorus							
0.78 (0.24 to 2.24)	mom+dm+hm						
0.63 (0.22 to 1.58)	0.81 (0.44 to 1.42)	mom+dm+bov					
0.62 (0.21 to 1.65)	0.8 (0.3 to 2.07)	0.99 (0.44 to 2.2)	form				
0.55 (0.19 to 1.4)	0.71 (0.36 to 1.35)	0.87 (0.55 to 1.42)	0.88 (0.4 to 2.02)	mom+form+bov			
0.46 (0.11 to 1.81)	0.58 (0.19 to 1.91)	0.73 (0.26 to 2.19)	0.72 (0.22 to 2.71)	0.82 (0.29 to 2.45)	mom		
0.39 (0.12 to 1.21)	0.51 (0.21 to 1.23)	0.63 (0.3 to 1.35)	0.64 (0.23 to 1.77)	0.72 (0.34 to 1.49)	0.87 (0.31 to 2.27)	mom+form	
0.38 (0.11 to 1.25)	0.5 (0.19 to 1.32)	0.62 (0.27 to 1.45)	0.62 (0.22 to 1.82)	0.7 (0.31 to 1.61)	0.86 (0.29 to 2.4)	0.98 (0.51 to 1.9)	mom+dm

Random effects model for classes

BIN - Unadjusted NMA (Odds Ratio)

phosphorus							
0.75 (0.24 to 2.34)	mom+dm+hm						
0.6 (0.21 to 1.81)	0.82 (0.29 to 2.22)	form					
0.6 (0.22 to 1.61)	0.81 (0.42 to 1.53)	1 (0.43 to 2.32)	mom+dm+bov				
0.54 (0.2 to 1.52)	0.73 (0.34 to 1.5)	0.89 (0.37 to 2.15)	0.89 (0.54 to 1.53)	mom+form+bov			
0.46 (0.11 to 1.88)	0.62 (0.18 to 2.16)	0.75 (0.2 to 2.81)	0.76 (0.25 to 2.28)	0.85 (0.29 to 2.53)	mom		
0.39 (0.11 to 1.46)	0.53 (0.18 to 1.61)	0.65 (0.2 to 2.13)	0.65 (0.28 to 1.73)	0.72 (0.29 to 1.88)	0.86 (0.27 to 2.88)	mom+dm	
0.39 (0.12 to 1.31)	0.52 (0.21 to 1.43)	0.64 (0.23 to 1.92)	0.64 (0.31 to 1.5)	0.72 (0.35 to 1.63)	0.86 (0.31 to 2.43)	0.99 (0.45 to 2.14)	mom+form

Time to Full Enteral Feed

Fixed effects model for interventions

CONT - Unadjusted NMA (Mean difference)

mom+dm+hdf														
-0.51 (-5.44 to 4.16)	mom+pform													
-0.56 (-5.73 to 4.41)	-0.01 (-2.18 to 1.98)	mom+pf+pdf												
-0.55 (-5.65 to 4.24)	-0.01 (-1.66 to 1.44)	-0.01 (-2.09 to 2.12)	mom+tform											
-0.85 (-5.29 to 3.7)	-0.33 (-4.56 to 4.23)	-0.31 (-4.86 to 4.7)	-0.31 (-4.7 to 4.54)	mom+dm										
-0.92 (-5.03 to 3.14)	-0.37 (-3.54 to 2.72)	-0.34 (-3.89 to 3.27)	-0.35 (-3.74 to 3.02)	-0.04 (-4.28 to 3.76)	mom+pf+pbov									
-1.07 (-6.07 to 3.78)	-0.59 (-2.08 to 0.9)	-0.55 (-2.97 to 1.97)	-0.55 (-2.58 to 1.58)	-0.24 (-5.03 to 4.19)	-0.2 (-3.64 to 3.2)	mom								
-1.27 (-5.48 to 2.84)	-0.72 (-3.98 to 2.5)	-0.67 (-4.46 to 2.92)	-0.67 (-4.28 to 2.71)	-0.39 (-4.75 to 3.48)	-0.27 (-1.34 to 0.4)	-0.14 (-3.67 to 3.39)	mom+pf+hp+pbov							
-1.71 (-6.11 to 2.81)	-1.2 (-5.43 to 3.3)	-1.16 (-5.78 to 3.79)	-1.16 (-5.52 to 3.65)	-0.86 (-1.75 to 0.03)	-0.8 (-4.61 to 3.41)	-0.6 (-5.03 to 4.18)	-0.46 (-4.36 to 3.88)	mom+dm+pbov						
-1.8 (-5.99 to 2.47)	-1.29 (-5.57 to 3.18)	-1.26 (-5.9 to 3.61)	-1.26 (-5.69 to 3.46)	-0.91 (-3.19 to 1.1)	-0.88 (-4.57 to 3.07)	-0.69 (-5.16 to 4.07)	-0.53 (-4.35 to 3.55)	-0.02 (-2.25 to 1.84)	mom+dm+ef					
-1.85 (-6.38 to 2.74)	-1.34 (-5.88 to 3.31)	-1.3 (-6.19 to 3.77)	-1.31 (-6 to 3.6)	-0.96 (-3.28 to 0.87)	-0.94 (-5 to 3.33)	-0.74 (-5.49 to 4.13)	-0.6 (-4.68 to 3.79)	-0.05 (-2.33 to 1.56)	-0.02 (-2.36 to 1.98)	mom+dm+lf+pbov				
-4.12 (-10.04 to 1.71)	-3.59 (-7.28 to 0.03)	-3.57 (-7.66 to 0.49)	-3.55 (-7.48 to 0.32)	-3.29 (-9.16 to 2)	-3.23 (-7.96 to 1.43)	-3.01 (-6.94 to 0.89)	-2.88 (-7.63 to 1.88)	-2.43 (-8.24 to 2.81)	-2.32 (-8.03 to 2.94)	-2.29 (-8.15 to 3.23)	mom+pf			
-4.98 (-9.57 to -0.29)	-4.4 (-8.47 to -0.4)	-4.38 (-8.77 to 0.05)	-4.37 (-8.57 to -0.15)	-4.04 (-9.01 to 0.49)	-4.05 (-7.19 to -0.81)	-3.84 (-7.99 to 0.46)	-3.71 (-6.93 to -0.25)	-3.2 (-8.08 to 1.27)	-3.13 (-7.87 to 1.37)	-3.08 (-8.02 to 1.67)	-0.81 (-6.1 to 4.53)	tform		
-5.02 (-9.38 to -0.7)	-4.45 (-8.21 to -0.84)	-4.43 (-8.55 to -0.3)	-4.4 (-8.38 to -0.59)	-4.09 (-8.86 to 0.06)	-4.1 (-6.86 to -1.35)	-3.9 (-7.82 to -0.01)	-3.75 (-6.66 to -0.84)	-3.25 (-7.92 to 0.86)	-3.18 (-7.74 to 0.89)	-3.12 (-7.88 to 1.31)	-0.89 (-5.88 to 4.21)	-0.02 (-1.86 to 1.6)	pform	

Random effects model for interventions

CONT - Unadjusted NMA (Mean difference)

mom+dm+hdf													
-0.49 (-5.37 to 4.37)	mom+pform												
-0.52 (-5.64 to 4.6)	-0.01 (-1.71 to 1.59)	mom+tform											
-0.57 (-5.76 to 4.71)	-0.01 (-2.15 to 2)	0 (-2.2 to 2.12)	mom+pf+pdf										
-0.78 (-5.4 to 3.95)	-0.35 (-4.61 to 4.59)	-0.31 (-4.86 to 4.86)	-0.29 (-4.89 to 4.91)	mom+dm									
-0.9 (-5.16 to 3.39)	-0.4 (-3.78 to 2.87)	-0.39 (-4.01 to 3.18)	-0.37 (-4.09 to 3.35)	-0.1 (-4.32 to 3.75)	mom+pf+pbov								
-1.1 (-6.42 to 4.13)	-0.59 (-2.69 to 1.41)	-0.54 (-3.18 to 2.03)	-0.55 (-3.35 to 2.3)	-0.24 (-5.6 to 4.41)	-0.19 (-4.03 to 3.66)	mom							
-1.19 (-5.55 to 3.23)	-0.71 (-4.3 to 2.7)	-0.67 (-4.49 to 2.98)	-0.65 (-4.6 to 3.11)	-0.38 (-4.71 to 3.64)	-0.19 (-1.51 to 0.69)	-0.11 (-4.1 to 3.91)	mom+pf+hp+pbov						
-1.62 (-6.11 to 3.09)	-1.21 (-5.46 to 3.78)	-1.17 (-5.67 to 4.02)	-1.14 (-5.72 to 4.1)	-0.85 (-2.04 to 0.29)	-0.72 (-4.57 to 3.36)	-0.61 (-5.32 to 4.75)	-0.47 (-4.45 to 3.84)	mom+dm+pbov					
-1.7 (-6.1 to 2.74)	-1.31 (-5.6 to 3.62)	-1.27 (-5.83 to 3.86)	-1.24 (-5.88 to 3.95)	-0.92 (-3.4 to 1.25)	-0.81 (-4.72 to 3.13)	-0.72 (-5.43 to 4.58)	-0.53 (-4.55 to 3.56)	-0.02 (-2.29 to 1.8)	mom+dm+ef				
-1.77 (-6.47 to 3.07)	-1.34 (-5.91 to 3.78)	-1.32 (-6 to 4.03)	-1.3 (-6.13 to 4.05)	-0.96 (-3.46 to 1.03)	-0.87 (-5.06 to 3.35)	-0.74 (-5.72 to 4.78)	-0.59 (-4.87 to 3.79)	-0.04 (-2.4 to 1.57)	-0.01 (-2.33 to 2.05)	mom+dm+lf+pbov			
-4.15 (-10.23 to 1.96)	-3.68 (-7.55 to 0.18)	-3.65 (-7.75 to 0.5)	-3.63 (-8 to 0.69)	-3.37 (-9.47 to 2.27)	-3.27 (-8.21 to 1.74)	-3.13 (-7.43 to 1.41)	-2.99 (-7.99 to 2.09)	-2.47 (-8.59 to 3.14)	-2.39 (-8.43 to 3.24)	-2.32 (-8.59 to 3.51)	mom+pf		
-4.94 (-9.55 to 0.13)	-4.39 (-8.6 to -0.17)	-4.37 (-8.86 to 0.1)	-4.35 (-8.92 to 0.28)	-4.13 (-8.96 to 0.68)	-4 (-7.42 to -0.5)	-3.79 (-8.37 to 0.83)	-3.72 (-7.22 to -0.01)	-3.29 (-8.15 to 1.53)	-3.19 (-7.96 to 1.62)	-3.15 (-8.13 to 1.92)	-0.68 (-6.44 to 4.94)	tform	
-4.99 (-9.34 to -0.24)	-4.44 (-8.3 to -0.6)	-4.42 (-8.56 to -0.35)	-4.4 (-8.71 to -0.17)	-4.18 (-8.84 to 0.27)	-4.04 (-7.07 to -1.04)	-3.86 (-8.13 to 0.45)	-3.74 (-6.92 to -0.59)	-3.33 (-7.89 to 1.15)	-3.24 (-7.78 to 1.27)	-3.18 (-7.9 to 1.54)	-0.73 (-6.24 to 4.57)	-0.01 (-1.92 to 1.66)	pform

Fixed effects model for classes

CONT - Unadjusted NMA (Mean difference)

mom+dm+hm							
-0.55 (-5.73 to 4.49)	mom+form						
-0.86 (-5.45 to 3.92)	-0.31 (-4.68 to 4.43)	mom+dm					
-1.1 (-5.55 to 3.37)	-0.52 (-4.08 to 2.95)	-0.22 (-4.64 to 3.73)	mom+form+bov				
-1.11 (-6.45 to 4.21)	-0.56 (-3.03 to 1.97)	-0.27 (-5.29 to 4.49)	-0.07 (-3.91 to 3.93)	mom			
-1.8 (-6.31 to 2.87)	-1.25 (-5.74 to 3.4)	-0.92 (-2.67 to 0.58)	-0.69 (-4.66 to 3.56)	-0.66 (-5.43 to 4.29)	mom+dm+bov		
-4.1 (-10.33 to 2.14)	-3.55 (-7.62 to 0.61)	-3.27 (-9.2 to 2.16)	-3.03 (-8.05 to 1.99)	-3 (-7.43 to 1.41)	-2.32 (-8.23 to 3.15)	mom+bov	
-4.98 (-9.75 to -0.18)	-4.4 (-8.53 to -0.32)	-4.06 (-8.98 to 0.37)	-3.9 (-7.12 to -0.54)	-3.85 (-8.21 to 0.59)	-3.14 (-7.9 to 1.32)	-0.85 (-6.2 to 4.59)	form

Random effects model for classes

CONT - Unadjusted NMA (Mean difference)

mom+dm+hm							
-0.53 (-5.94 to 4.72)	mom+form						
-0.78 (-5.49 to 4.21)	-0.32 (-4.73 to 4.79)	mom+dm					
-1.03 (-5.6 to 3.59)	-0.53 (-4.33 to 3.11)	-0.23 (-4.6 to 3.72)	mom+form+bov				
-1.09 (-6.85 to 4.54)	-0.57 (-3.48 to 2.33)	-0.23 (-5.84 to 4.54)	-0.04 (-4.35 to 4.32)	mom			
-1.7 (-6.38 to 3.06)	-1.26 (-5.73 to 3.83)	-0.92 (-2.9 to 0.85)	-0.65 (-4.7 to 3.55)	-0.69 (-5.59 to 4.89)	mom+dm+bov		
-4.1 (-10.56 to 2.42)	-3.62 (-7.99 to 0.73)	-3.33 (-9.63 to 2.43)	-3.1 (-8.32 to 2.22)	-3.11 (-7.85 to 1.9)	-2.36 (-8.66 to 3.4)	mom+bov	
-4.94 (-9.74 to 0.14)	-4.38 (-8.68 to -0.17)	-4.17 (-8.92 to 0.53)	-3.89 (-7.32 to -0.25)	-3.84 (-8.55 to 0.91)	-3.22 (-7.98 to 1.46)	-0.74 (-6.45 to 5.05)	form

Bayley Scales II MDI

Fixed effects model for interventions

CONT - Unadjusted NMA (Mean difference)

mom+pf+pbov						
0.8 (-6.66 to 8.55)	pform					
1.31 (-2.85 to 5.42)	0.52 (-6.89 to 7.55)	mom+pf+pdf				
1.46 (-2.65 to 5.58)	0.67 (-5.75 to 6.98)	0.04 (-3.45 to 4.07)	mom+pform			
2.73 (-1.61 to 7.13)	1.98 (-6.72 to 10.47)	1.42 (-4.49 to 7.54)	1.3 (-4.64 to 7.4)	mom+dm+pbov		
2.9 (-3.09 to 8.71)	2.08 (-5.85 to 9.66)	1.57 (-4.04 to 7.34)	1.45 (-3.14 to 5.93)	0.21 (-7.21 to 7.43)	mom+dm	
3.52 (-4.56 to 11.68)	2.79 (-8.19 to 13.47)	2.26 (-6.57 to 11.33)	2.06 (-6.75 to 11.29)	0.84 (-6.46 to 8.2)	0.57 (-9.16 to 10.93)	mom+dm+hdf

Random effects model for interventions

CONT - Unadjusted NMA (Mean difference)

mom+pf+pbov						
0.8 (-6.76 to 8.17)	pform					
1.3 (-3.14 to 5.7)	0.45 (-6.74 to 7.88)	mom+pf+pdf				
1.36 (-2.8 to 5.68)	0.55 (-5.72 to 7.25)	0.03 (-3.66 to 4.25)	mom+pform			
2.7 (-2.2 to 7.24)	1.83 (-7.11 to 10.97)	1.33 (-5.16 to 7.97)	1.25 (-5.21 to 7.61)	mom+dm+pbov		
3.33 (-4.66 to 11.8)	2.57 (-8.62 to 13.97)	1.97 (-7.01 to 11.57)	1.93 (-7.24 to 11.46)	0.72 (-6.27 to 8.16)	mom+dm+hdf	
2.86 (-3.51 to 9.45)	2.03 (-6.06 to 10.2)	1.57 (-4.81 to 7.68)	1.47 (-3.71 to 6.5)	0.28 (-7.85 to 8.42)	-0.51 (-11.09 to 9.82)	mom+dm

Fixed effects model for classes

CONT - Unadjusted NMA (Mean difference)

mom+form+bov					
0.81 (-7.12 to 8.88)	form				
1.34 (-3.08 to 5.78)	0.57 (-7.04 to 8.11)	mom+form			
2.71 (-2.83 to 7.87)	1.95 (-7.78 to 11.35)	1.31 (-5.68 to 8.21)	mom+dm+bov		
2.86 (-3.85 to 9.4)	2.05 (-6.95 to 10.89)	1.46 (-4.72 to 7.93)	0.14 (-8.31 to 8.49)	mom+dm	
3.45 (-5.18 to 12.11)	2.68 (-9.01 to 14.1)	2.07 (-7.31 to 11.95)	0.79 (-7.67 to 9.22)	0.51 (-9.99 to 11.8)	mom+dm+hm

Random effects model for classes

CONT - Unadjusted NMA (Mean difference)

mom+form+bov					
0.73<br>(-7.11 to 8.77)	form				
1.32<br>(-3.21 to 5.88)	0.53<br>(-7.17 to 8.15)	mom+form			
2.61<br>(-3.25 to 8.1)	1.9<br>(-8.25 to 11.68)	1.19<br>(-6.15 to 8.65)	mom+dm+bov		
2.79<br>(-4.29 to 9.94)	1.99<br>(-7.38 to 11.11)	1.5<br>(-5.3 to 7.91)	0.29<br>(-9.01 to 9.54)	mom+dm	
3.28<br>(-5.3 to 12.12)	2.56<br>(-9.34 to 14.56)	1.92<br>(-7.64 to 11.98)	0.7<br>(-7.58 to 9.61)	0.5<br>(-10.53 to 11.86)	mom+dm+hm
